# Topical Probiotics as a Novel Approach in the Treatment of Chronic Dermatoses Associated with Skin Dysbiosis: A Narrative Review

**DOI:** 10.3390/ijms262010195

**Published:** 2025-10-20

**Authors:** Danuta Nowicka, Emilia Kucharczyk, Karolina Pawłuszkiewicz, Matylda Korgiel, Tomasz Busłowicz, Małgorzata Ponikowska

**Affiliations:** 1 University Centre of General Dermatology and Oncodermatology, Department of Aesthetic Dermatology and Skin Regenerative Medicine, Faculty of Medicine, Wroclaw Medical University, ul. Borowska 213, 50-556 Wrocław, Poland; danuta.nowicka@umw.edu.pl; 2 Faculty of Medicine, Wroclaw Medical University, Wybrzeże L. Pasteura 1, 50-367 Wrocław, Poland; emilia.kucharczyk@student.umw.edu.pl (E.K.); karolina.pawluszkiewicz@student.umw.edu.pl (K.P.); matylda.korgiel@student.umw.edu.pl (M.K.); tomasz.buslowicz@student.umw.edu.pl (T.B.); 3 University Centre of General Dermatology and Oncodermatology, Faculty of Medicine, Wroclaw Medical University, ul. Borowska 213, 50-556 Wrocław, Poland

**Keywords:** skin, inflammation, oxidative stress, natural compounds, inflammaging, topical probiotics, microbiota, immunomodulation

## Abstract

The skin microbiome plays a pivotal role in maintaining skin homeostasis, immune regulation, and barrier integrity. Dysbiosis, characterized by altered diversity and function of the microflora, contributes to the pathogenesis of chronic inflammatory dermatoses such as atopic dermatitis, psoriasis, acne vulgaris, hidradenitis suppurativa, rosacea, and photoaging. This narrative review, based on searches in PubMed, Scopus, and Google Scholar, summarizes current evidence on the role of topical probiotics in the prevention and management of inflammatory dermatoses, drawing mainly on studies from the past decade and, where relevant, earlier works published between 1975 and 2025. Evidence indicates that topical probiotics modulate local immune responses, enhance antimicrobial peptide synthesis, inhibit pathogenic microorganism colonization, and support skin barrier regeneration. Additional benefits include accelerated wound healing and reduced environmental damage. However, study results are heterogeneous, and designs vary, with limited data on long-term effects, particularly in paediatric and immunosuppressed populations. Topical probiotics are a promising therapeutic approach for chronic inflammatory dermatoses linked to microbiota dysbiosis. They can restore microbial balance, support barrier function, suppress pathogenic microorganisms, and promote skin regeneration. Despite consistent reports of clinical improvement and improved cutaneous defence mechanisms, small sample sizes, methodological heterogeneity, and the absence of standardized dosing regimens limit current evidence. Long-term safety data are limited, especially for vulnerable patient groups. Rigorous randomized controlled trials with standardized protocols and larger, diverse populations are needed to confirm efficacy, ensure safety, and guide clinical implementation.

## 1. Introduction

The skin is the body’s primary barrier against ultraviolet radiation, physicochemical insults, microbial invasion, and transepidermal water loss (TEWL). It consists of the epidermis—composed mainly of keratinocytes forming the stratum corneum—and the dermis, a fibrous connective tissue rich in extracellular matrix components such as collagen, elastin, fibronectin, and proteoglycans, which collectively maintain mechanical strength and structural cohesion across skin layers [1,2]. Beyond its role as a mechanical barrier, the skin actively participates in both innate and adaptive immune responses, contributing to immunological surveillance, pathogen recognition, and inflammatory regulation [2].

The skin harbours a diverse microbiota that plays a crucial role in maintaining cutaneous health by regulating immune responses, preserving barrier integrity, protecting against pathogenic microorganisms, and modulating local inflammation. The skin microbiota, which comprises a diverse consortium of microorganisms—including bacteria, fungi, viruses, and mites—is broadly classified into resident (commensal) organisms, which establish stable, mutually beneficial relationships with the host, and transient (potentially pathogenic) species, which are acquired from the environment and may disrupt microbial equilibrium under certain conditions [3,4,5]. Its composition is influenced by factors such as anatomical site, humidity, age, genetic background, and immune status [3,4,5,6].

Following the potential involvement in the pathogenesis of skin diseases, alterations in the composition or function of commensal microbiota have become a significant subject of contemporary research. Dysbiosis is increasingly associated with chronic inflammatory dermatoses, including atopic dermatitis (AD), psoriasis, acne vulgaris, rosacea, and others [7,8]. Scientific studies provide reliable evidence of changes in the abundance of specific microorganisms within the overall microbial population or complete shifts in its composition. Dysbiotic changes are not only a reflection of pathological processes but constitute an integral component of their pathogenesis and, depending on their severity, may significantly affect the clinical picture.

The scientific community has shown increasing interest in the therapeutic potential of microbiome-modulating agents—collectively termed biotics—for the management of dysbiosis-related dermatoses. This group encompasses: probiotics, which are preparations containing live microorganisms aimed at restoring commensal microbiota balance (1); prebiotics, defined as selectively metabolized substrates that promote the growth and activity of beneficial microbes (2); postbiotics, consisting of non-viable microbial cells, fragments, or metabolites—such as short-chain fatty acids, lysates, or anti-inflammatory proteins—that exert direct effects on the host skin (3); and synbiotics, which are strategic combinations of probiotics and prebiotics designed to enhance their synergistic action [9,10,11].

Previous reviews on the use of topical probiotics have most often presented this therapy in a general manner, combining cosmetic aspects, wound healing, and inflammatory skin diseases. Although they provide valuable information, they rarely offer an in-depth analysis of individual dermatoses or address specific pathogenetic pathways and patterns of dysbiosis. Our article fills this gap by focusing on chronic inflammatory dermatoses, compiling the latest clinical and experimental data, and discussing issues of standardization and priorities for further research. In this context, we examine the emerging role of topical probiotics as a promising, yet still underexplored, therapeutic approach, providing an overview of the immunological functions of the skin microbiome and the potential of its modulation to restore microbial homeostasis and influence immune pathways relevant to disease pathogenesis.

## 2. The Skin Microbiome and Its Role in Inflammatory Skin Disease

### 2.1. The Healthy Skin Microbiome: Composition, Function, Environmental Influence

The skin microbiota is divided into commensal microorganisms, which make up the majority of its composition and maintain a stable, symbiotic relationship with the host (resident microbiota), and pathogenic microorganisms, which represent a smaller proportion and opportunistically colonize the skin from the external environment, forming the transient microbiota [3,4,5]. Although the skin’s external surface area in adults is typically estimated at approximately 2 m^2^, inclusion of its appendages—such as hair follicles and the ducts of sweat and sebaceous glands—reveals a significantly expanded interface, exceeding 30 m^2^, that facilitates extensive microbial interaction [12]. The bacterial load on the skin ranges from 10^4^ to 10^6^ microorganisms per cm^2^, with more than 200 distinct genera identified to date [13].

The skin microbiota comprises bacteria, fungi, viruses, and skin mites [6]. The four main bacterial phyla identified on the skin are Actinobacteria (52%), Firmicutes (24%), Proteobacteria (17%), and Bacteroidetes (7%) [6]. Among the predominant skin commensal microorganisms are coagulase-negative staphylococci—most notably *Staphylococcus epidermidis*—as well as anaerobic *Cutibacterium acnes* (formerly *Propionibacterium acnes*), *Micrococcus*, *Streptococcus*, *Corynebacterium*, and *Acinetobacter* species [6,13,14,15]. The viral component of the cutaneous microbiome is dominated by bacteriophages, which modulate bacterial communities through their lytic activity and contribute to microbial homeostasis [16]. Byrd et al. (2018) identified the ten most common skin-associated viruses, including various phages, Alphapapillomaviruses, β-, γ-, and μ-human papillomaviruses, Merkel cell polyomavirus, Molluscum contagiosum virus, HPyV6 and HPyV7 polyomaviruses, RD114 retrovirus, and Simian virus [16]. Notably, papillomaviruses and Molluscum contagiosum can cause cutaneous lesions, while Merkel cell polyomavirus is associated with carcinogenesis [5,17]. Fungal skin commensals include *Malassezia*, *Cryptococcus*, *Rhodotorula*, and *Candida* species, with *Malassezia* being predominant, especially on seborrheic areas like the trunk and arms, while feet harbour greater fungal diversity [12]. *Demodex* mites—*D. folliculorum* and *D. brevis*—inhabit sebaceous regions, including the face, eyelids, and nasal alae [3,5,17,18,19]. Their microbiota has been linked to dermatologic conditions such as papulopustular rosacea [20]. *Micrococcus luteus* is one of the most prevalent species in the human cutaneous microbiota, predominantly colonizing the head, legs, and arms. It exhibits broad-spectrum antimicrobial activity, contributing to the control of conditions such as acne, eczema, psoriasis, impetigo, athlete’s foot, methicillin-resistant *Staphylococcus aureus* (MRSA), and body odour. It enhances the protective functions of the skin microbiota [21]. *Candida albicans* resides on cutaneous-mucosal surfaces, including the oral cavity, skin, female reproductive tract, and gastrointestinal system. While typically a commensal yeast in healthy individuals, it may become opportunistic under immunosuppression, microbial imbalance, or epithelial barrier disruption [22].

The skin microbiome varies significantly across different anatomical sites, which is presented in Table 1 [3,6,19,23,24,25].

Colonization of the human microbiome begins at birth, with the mode of delivery playing a pivotal role in determining the initial microbial communities [16,26]. As individuals age, the skin microbiota evolves under the influence of both intrinsic and extrinsic factors, leading to considerable inter- and intrapersonal variability.

Despite this diversity, a core set of microbial species is consistently shared across individuals. Interpersonal differences are largely attributed to rare or transient species shaped by lifestyle, environmental exposures, and genetic predisposition [3]. Intrapersonal variation is particularly noticeable on sites such as the forearms and palms, where unique microenvironments, hygiene practices, and the use of cosmetic products—like hand creams—contribute to microbial diversity [27]. Additionally, sex-related differences in microbiota composition have been identified in specific regions, such as the axilla [19,28].

**Figure 1:**
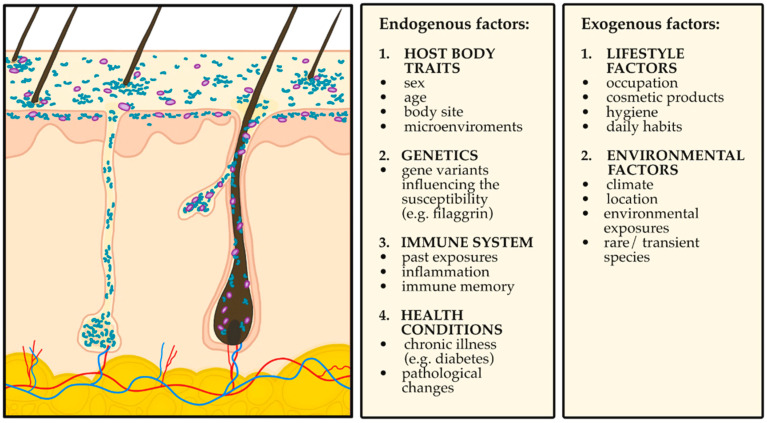
Endogenous and Exogenous Factors Influencing the Composition of the Cutaneous Microbiome [3,19,27,28].

Figure 1 presents the main intrinsic and extrinsic factors shaping the composition and diversity of the cutaneous microbiome [3,19,27,28].

The skin microbiome plays an important role in maintaining cutaneous health by participating in regulating immune responses, supporting barrier integrity, and protection against pathogens.

The maintenance of skin homeostasis is supported by microbial metabolic activities. These include the secretion of proteolytic enzymes, which ease the desquamation process and contribute to the renewal of the stratum corneum [29]. Additionally, microbial production of sebum and free fatty acids plays a pivotal role in regulating the skin’s pH [29]. Lipase enzymes are involved in the breakdown of the superficial lipidic film, affecting barrier integrity and microbial colonization dynamics [30,31]. Urease enzymes contribute to urea degradation, further influencing nitrogen metabolism [30,31]. *Staphylococcus epidermidis* supports skin barrier integrity by producing sphingomyelinase and promoting ceramide synthesis, a significant lipid in the epithelial barrier [32]. Commensal microorganisms are also involved in biofilm formation and bacteriocin secretion, and they collectively contribute to microbial community regulation and stability [30,31].

The cutaneous microbiota also provides protection against potential pathogens through competitive exclusion [33,34] and the production of antimicrobial peptides (AMPs) by commensal bacteria [35,36]. *Malassezia* species secrete indole compounds that inhibit the growth of various yeasts and molds [37]. The cutaneous commensal flora interacts synergistically with various innate immune mechanisms, including complement proteins and AMPs [38]. AMPs (such as human β-defensin (HBD-2), cathelicidins, and LL-37) and other bioactive proteins secreted by skin-resident microbes play a pivotal role in modulating cutaneous immunity. These molecules stimulate keratinocytes via pattern recognition receptors (PRRs), particularly Toll-like receptor 2 (TLR-2), leading to the upregulation of additional AMPs, cytokines, and chemokines in response to microbial proliferation [3,39,40]. This immunological cascade supports the maintenance of microbial balance and reduces dysbiosis [3]. Furthermore, skin appendages such as the pilosebaceous units and eccrine glands possess intrinsic immunomodulatory capabilities [41]. Pilosebaceous units respond to stimulation by Gram-positive bacteria by producing AMPs, while their lipid-rich environment—especially due to the presence of free fatty acids—also plays a significant role in inhibiting microbial growth. In contrast, eccrine sweat glands secrete dermcidin, a relatively weak AMP, which nonetheless contributes to the regulation of the cutaneous microbial ecosystem [42]. Another defensive pathway activated by skin cells in response to bacterial pathogens involves PRRs. One such intracellular PRR, nucleotide-binding oligomerization domain-containing protein 2 (NOD2), detects bacterial peptidoglycans derived from both Gram-positive and Gram-negative organisms. Experimental disruption of NOD2 has been shown to result in microbial imbalance and localized alterations in AMP expression [43]. Although the exact mechanisms remain to be fully clarified, current evidence indicates that NOD2 plays a key role in immune surveillance and functions in coordination with the cutaneous microbiota to shape host defence responses. Importantly, this system allows commensal microorganisms to recognize potentially pathogenic microbes and initiate an innate immune response. Nucleotide-binding oligomerization domain containing 2 (NOD2) in cooperation with Toll-like receptors (TLR)—TLR2 and TLR6 offers enhanced protection against *Staphylococcus aureus*, while TLR2, TLR3, TLR7, TLR8, and TLR9 collectively safeguard the skin from viral infections such as those caused by herpesviruses, papillomaviruses, and poxviruses [44].

**Figure 2:**
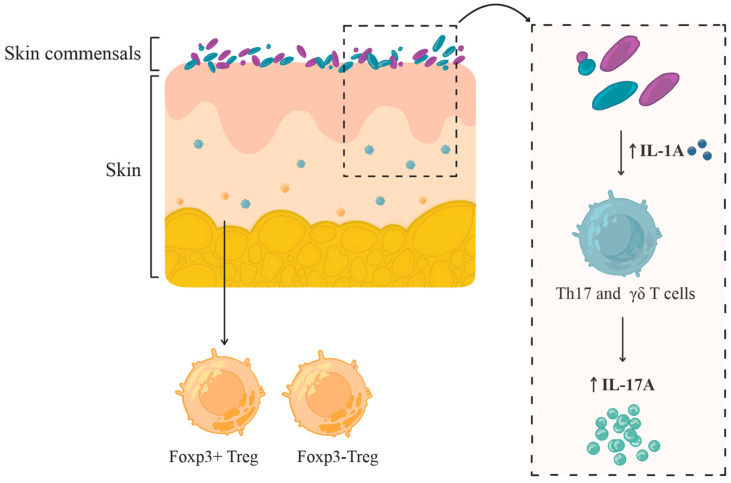
Immunomodulatory Role of Skin Commensals in Cytokine Regulation and Treg Cell Differentiation [38,43,44,45,46]. Skin-resident commensal microorganisms actively regulate local cytokine signalling by modulating interleukin-1 (IL-1) expression. Elevated IL-1 levels subsequently stimulate the release of interleukin-17A (IL-17A) and interferon-γ (IFN-γ) by dermal T helper 17 cells (Th17) and γδ T cells. Regulatory T cells (Tregs), predominantly located near hair follicles, engage with commensals during a defined postnatal window to establish immune tolerance. Both classical Foxp3^+^ Tregs and non-classical Foxp3^−^ Treg subsets contribute to this process.

Figure 2 illustrates the immunomodulatory functions of skin commensals, particularly their involvement in cytokine regulation and regulatory T cells (Treg) differentiation crucial for immune homeostasis [38,43,44,45,46].

Stimulation of cytokine production by skin commensals has been demonstrated in the work of Naik et al. (2015) in Table 2 [46].

Commensal skin microbes are closely connected with the immune system and may influence wound healing; however, current evidence remains unproven. While some studies report accelerated wound closure in the absence of microbiota [47], others highlight beneficial roles of *Staphylococcus epidermidis* in promoting unconventional repair through regulatory CD8+ T cell recruitment [48], aligning with findings on the positive impact of a balanced microbiota [49].

### 2.2. Microbiome Alterations in Atopic Dermatitis (AD)

Atopic dermatitis (AD) is a chronic, pruritic skin disease with recurrent eczematous lesions, typically starting in infancy and primarily affecting children [50]. While it often resolves in childhood, severe cases may persist into adulthood or reappear later in life [51].

#### 2.2.1. The Role of *Staphylococcus aureus* in AD

*Staphylococcus aureus*, a Gram-positive opportunistic pathogen, is also implicated in both superficial and deep-seated skin infections. It produces a broad spectrum of virulence factors—such as phenol-soluble modulins (PSMs), exotoxins, proteolytic enzymes, and superantigens including toxic shock syndrome toxin-1 (TSST-1) and enterotoxins (e.g., SEB, SEA)—that collectively exacerbate cutaneous inflammation and compromise epidermal integrity [52,53,54,55,56]. A key feature of AD is an imbalance in the skin microbiome, often characterized by an increased dominance of *Staphylococcus aureus*—both in relative abundance and absolute quantity [5,57]. *S. aureus* colonizes lesional and non-lesional skin and suppresses the growth of commensal bacteria such as *Corynebacterium* and *S. epidermidis* [58]. This dysbiosis results in reduced microbial diversity—though overall richness appears to be less affected—and contributes to impaired skin barrier function, heightened inflammation, and an increased susceptibility to skin infections [5,59,60,61]. A high abundance of *S. aureus* is strongly correlated with enhanced type 2 immune reaction, increased allergen sensitization, and greater severity of AD [62]. This association has been consistently validated across studies and appears to be influenced by host-specific factors such as sex, age, and ethnicity [61,63,64]. A-toxin and δ-toxin secreted by *Staphylococcus aureus* promote the degradation of AMPs, thereby compromising the skin’s innate immune defences [16,60]. Moreover, immune responses driven by Th2 and Th17 cells further suppress AMP production, exacerbating microbial dysbiosis and enabling persistent colonization of AD lesions by *S. aureus* [58].

An impaired skin barrier in atopic dermatitis (AD) facilitates *Staphylococcus aureus* colonization, partly due to altered stratum corneum architecture and increased fibronectin exposure that enhances bacterial adhesion [65]. Filaggrin (FLG) ensures proper keratin alignment within corneocytes, while its degradation products serve as natural moisturizing factors (NMFs), both critical for skin hydration and barrier integrity. FLG loss-of-function mutations, which are a major genetic risk factor of AD in Asian and Caucasian populations, reduce levels of acidic breakdown products like urocanic acid and pyrrolidone carboxylic acid, leading to elevated skin pH, which favours *S. aureus* growth and upregulation of adhesion and immune evasion proteins (e.g., clumping factor B, fibronectin-binding proteins) [66,67,68].

#### 2.2.2. Non-*S. aureus*-Related Microbial Changes in AD

*Corynebacterium tuberculostearicum* has been found to proliferate during AD flare-ups, with genomic evidence indicating the presence of multiple potential virulence factors [69]. Moreover, an increased abundance of other *Staphylococcus* species, including *S. capitis* and *S. lugdunensis*, has been reported in individuals with severe atopic dermatitis (AD) [70].

In AD skin fungi and viruses also exhibit site-specific alterations [71]. *Malassezia* species, especially *M. globosa* and *M. restricta*, dominates both healthy and AD skin [72,73]. However, in individuals with AD, elevated skin pH facilitates overexpression of *Malassezia*-derived allergens, potentially enhancing inflammatory responses [74,75]. Increased relative abundances of *M. dermatitis* and *M. sympodialis* have been reported in individuals with a history of AD, although not necessarily during active disease phases [59,72,73]. *Malassezia*-specific immunoglobulins E (IgE) are frequently elevated in patients with AD and have been positively correlated with disease severity [76,77]. *M. sympodialis* exacerbates inflammation via the release of extracellular vesicles capable of inducing interleukin-4 (IL-4) and tumour necrosis factor alpha (TNF-α), promoting degranulation and cysteinyl leukotriene secretion in IgE-sensitized mast cells [78,79]. Furthermore, *M. globosa* secretes the MGL_1304 protein, which has been identified in the sweat of AD patients and is implicated in type I hypersensitivity reactions [78]. *Candida albicans* has been shown to be significantly enriched in lesional cheek skin of AD patients, with a 100% detection rate compared to 10% in healthy controls [80]. Despite these insights, the currently limited data on cutaneous eukaryotic communities in atopic dermatitis highlights the necessity for larger, well-designed studies to confirm and expand upon these findings.

The skin virome, composed of diverse RNA and DNA viruses, represents another underexplored component of the cutaneous microbiome. Nevertheless, bacteriophages are recognized as potent modulators of microbial homeostasis in AD patients through lytic activity and horizontal gene transfer [17].

However, the current evidence is largely derived from cross-sectional studies with heterogeneous methodologies, making it difficult to establish causality between dysbiosis and disease activity. Future research should focus on longitudinal and interventional designs, employ standardized sampling and sequencing protocols, and explore the impact of microbiome modulation on both clinical outcomes and molecular inflammatory pathways. Based on 16S rRNA sequencing, Table 3 summarizes a few studies that describe changes in the microbiome in atopic dermatitis (AD) [58,70,73,81,82,83]. The table was prepared based on a PubMed search using the terms (atopic dermatitis) AND (16S rRNA sequencing) conducted on 20–21 September 2025. Only studies with full open access to the article and involving human subjects were included, while animal studies were excluded. In addition, studies previously cited in the text as summaries of microbiota alterations in AD were also incorporated.

### 2.3. Microbiome Alterations in Psoriasis

Psoriasis is a chronic immune-mediated inflammatory disease (IMID) dermatosis marked by sustained cutaneous inflammation and aberrant keratinocyte proliferation [84]. The precise pathogenesis remains incompletely understood, though it is postulated that environmental stimuli initiate a T-cell-driven immune cascade, leading to epidermal hyperplasia. Recognized triggers include mechanical trauma (Koebner phenomenon), ultraviolet injury, HIV, streptococcal infections (notably in guttate psoriasis), certain pharmacologic agents (e.g., beta-blockers, ACE inhibitors), psychological stress, alcohol use, tobacco exposure, and obesity [85]. Smoking is a recognized risk factor for both the initiation and persistence of psoriasis, and it may impair therapeutic efficacy [86]. Although no direct studies have addressed its impact on the skin microbiome in psoriasis [87], tobacco smoke is known to disrupt the lung and gut microbiota, reducing microbial diversity and promoting genera such as *Bacteroides*, *Prevotella*, *Enterobacteriaceae*, and *Clostridium*. Emerging evidence suggests that alterations in the gut microbiome may also be relevant to psoriasis, supporting the concept of a gut–skin axis. Specific microbial signatures have been associated with disease severity and could potentially influence the clinical response to biologic therapies [88].

As previously discussed, the skin microbiome significantly influences Th17-mediated immunity—a central pathway in psoriasis pathogenesis. This suggests that the microbiome may play a role in disease development. However, previous studies have presented inconclusive results, making it difficult to establish a clear causal relationship between microbial dysbiosis and psoriasis [89].

Additional studies have underscored the involvement of *Candida albicans* in psoriasis pathogenesis. Dendritic cells, upon recognition of fungal β-glucan, trigger interleukin 36 alpha (IL-36α) secretion, promoting psoriasiform inflammation [90]. Notably, the M protein of *Streptococcus pyogenes*, frequently found on psoriatic skin, exhibits molecular mimicry with 50-kDa type I keratin. This mimicry activates autoreactive T cells, initiating cutaneous inflammation and psoriatic lesion development [91,92].

Table 4 provides an overview of selected studies reporting microbiome alterations in psoriatic skin based on 16S rRNA sequencing [86,93,94,95,96,97,98,99,100,101]. The table was prepared based on a PubMed search using the terms (psoriasis) AND (16S rRNA sequencing) conducted on 20–21 September 2025. Only studies with full open access to the article and involving human subjects were included, while animal studies were excluded.

The collection of samples from both lesional and non-lesional skin across heterogeneous anatomical sites introduces significant variability in the cutaneous microbiota composition [20]. This variability is further influenced by the sampling methods, including both superficial and invasive approaches—such as swabs and biopsies, respectively—which access different layers of the skin, each harbouring distinct microbial communities [98].

While current studies frequently rely on varied methods such as skin swabs, tape stripping, scraping or biopsies, no universally accepted protocol for microbiome sampling in dermatological research has yet been established [102,103]. Differences in pressure, swab material, sampling direction, or anatomical depth across methods contribute to inconsistencies in microbial recovery and comparability between studies [103].

Recently, efforts such as the protocol proposed by Perugini et al. (2023) [103] have begun to address this issue by introducing a reproducible and non-invasive approach integrating biophysical measurements with microbiota sampling under controlled conditions. This protocol demonstrates that, with standardized questionnaires, defined anatomical sites, and consistent lifestyle monitoring, interindividual variability can be minimized and microbial data made more reliable [103].

However, despite such advances, the need for standardized sampling guidelines remains [102]. Future frameworks should consider variables such as location, climate, cosmetic use, region and account hygiene regimen [104], to enhance consistency and enable reliable cross-study comparisons.

## 3. Topical Probiotics in the Treatment of Chronic Dermatoses

Probiotics are “live microorganisms that, when administered in adequate amounts, confer a health benefit on the host”—a definition reaffirmed by the International Scientific Association for Probiotics and Prebiotics (ISAPP) to promote precise scientific and clinical use [105]. Although they have been traditionally used to support gut health, their use in dermatology is getting more attention. When applied directly to the skin, probiotics or their byproducts can change the local microbiome, decrease inflammation, combat pathogenic bacteria and promote skin barrier repair [10,106,107,108,109].

**Figure 3:**
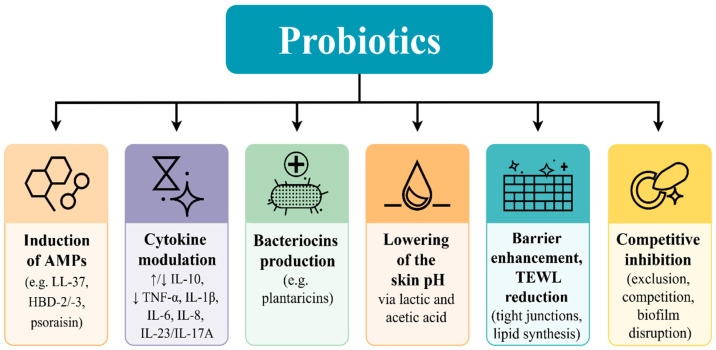
Modes of Action of Topically Applied Probiotics [110,111,112,113,114,115,116,117,118,119,120,121,122]. Abbreviations: AMPs—Antimicrobial peptides; HBD-2—Human beta-defensin; IL—Interleukin; TNF-α—Tumor necrosis factor alpha; TEWL—Transepidermal water loss.

An overview of the modes of action of topically applied probiotics, which render them beneficial in various skin diseases, is outlined in Figure 3 [110,111,112,113,114,115,116,117,118,119,120,121,122].

### 3.1. Mechanistic Insights into Topical Probiotics: Immunomodulation, Pathogen Inhibition, and Barrier Support

Topical probiotics exert their skin protective action through a complex cascade of immunological, microbiological, and biochemical interactions that cumulatively suppress inflammation, restore microbial balance, and enhance barrier integrity [108,109,121].

**Figure 4:**
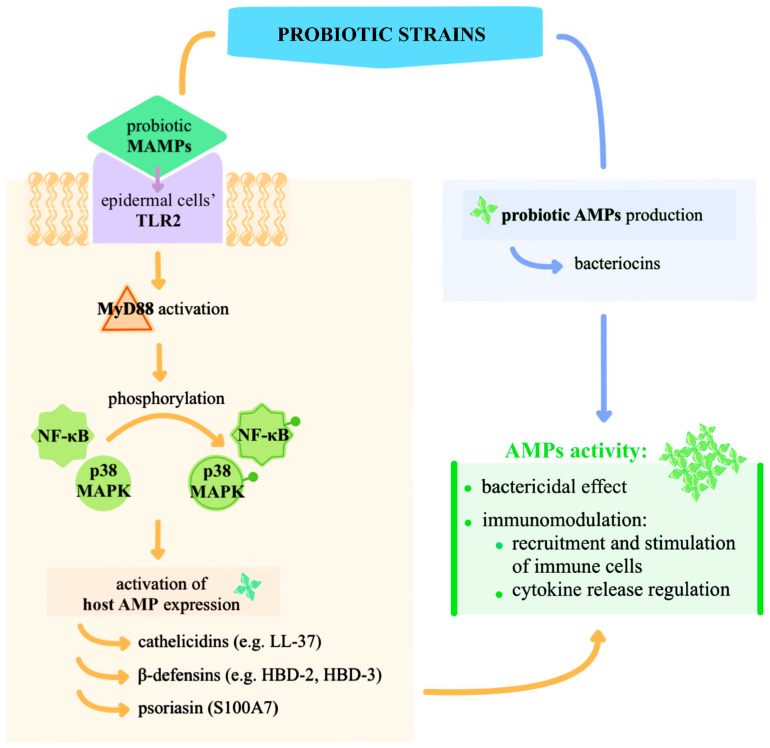
Mechanistic Pathway of Probiotic-Induced Skin Immunomodulation. The mechanism is initiated by the binding of probiotics to PRRs, particularly TLR2, which is densely expressed on keratinocytes and Langerhans cells of the epidermis [108,122,123]. Through binding to microbial-associated molecular patterns (MAMPs) of probiotic bacteria such as *Lactobacillus* spp. and *Bifidobacterium* spp., TLR2 activates downstream intracellular signalling cascades such as the myeloid differentiation primary response 88 (MyD88) adaptor protein, leading to the phosphorylation of nuclear factor kappa-light-chain-enhancer of activated B cells (NF-κB) and mitogen-activated protein kinases (MAPKs) such as p38 MAPK [124,125]. This activation cascade results in the transcriptional activation of host-derived AMPs [122,126]. These include human-derived peptides such as cathelicidins (e.g., LL-37), human β-defensins 2 and 3 (HBD-2, HBD-3), and psoriasin (S100A7), which are components of the innate immune defence of the skin [122,123,124,126]. Probiotic strains themselves can also produce AMPs, including bacteriocins, which have direct activity against skin pathogens [108]. Besides their bactericidal effect, AMPs are also immune modulators: they recruit and stimulate immune cells like neutrophils, macrophages, and T cells and regulate cytokine release to modulate inflammation [122,123,126].

Figure 4 illustrates the molecular pathway through which probiotics modulate skin immunity and enhance host defence mechanisms.

### 3.2. Experimental Validation of Immunomodulation of Probiotics Applied Topically

Growing evidence from in vitro models and clinical trials supports the immunomodulatory properties of topically applied probiotics, which can enhance innate immune responses, reduce pathogen colonization, and restore skin barrier integrity [109,122,124,125].

In an early in vitro model, Rosignoli et al. (2018) [122] applied heat-treated *Lactobacillus johnsonii* (HT La1) to a reconstructed human epidermis and noted a significant upregulation of AMP gene expression and protein production, including an increase in HBD-2 and a 74% reduction in *Staphylococcus aureus* adhesion to keratinocytes [109,122]. This indicates that probiotics not only potentiate the host’s innate immune responses but also actively reduce pathogenic bacterial colonization through competitive exclusion and modulation of the skin microenvironment.

Additionally, the study conducted by Lebeer et al. (2022) [125] showed that live *Lactobacillus* strains, applied topically, significantly reduced inflammatory acne lesions in a placebo-controlled trial. The probiotic cream was well tolerated and improved skin hydration by over 37% after two weeks and 45% after four weeks—which is particularly valuable in acne management, where many treatments tend to dry out the skin [124]. While the exact immunomodulatory mechanisms remain unclear, these results support the potential of live probiotics as a safe and effective skin therapy [125].

Further providing evidence for the immunomodulatory effects of topical probiotics, clinical studies with *Vitreoscilla filiformis* lysate in patients with Atopic Dermatitis (AD) have shown reduced *S. aureus* colonization, diminished inflammation, and improved skin barrier function. Mechanistically, *V. filiformis* lysate was found to stimulate TLR2-mediated innate immune responses, induce the anti-inflammatory cytokine interleukin-10 (IL-10), promote T-regulatory cell activity, and increase TNFAIP3/A20 expression—a potent negative regulator of inflammation—in keratinocytes [126,127]. These findings highlight the ways in which microbial components can modulate immune responses and enhance cutaneous homeostasis, even in the context of chronic inflammatory disease, such as AD.

Supporting this, Nguyen et al. (2023) [128] demonstrated that a fermented lysate of *L. plantarum* K8 significantly enhanced HBD-2 and HBD-3 expression via p38 MAPK and NF-κB signalling and suppressed interleukin-6 (IL-6), interleukin-8 (IL-8), TNF-α, and interleukin-1-beta (IL-1β) levels in an *S. aureus*-induced mouse ear erythema model [128]. This corroborates the fact that probiotics and their metabolites can act as AMP inducers and cytokine modulators in both prophylactic and therapeutic interventions [10,108].

Although the imiquimod-induced psoriasis-like mouse model study by Chen et al. (2017) administered *Lactobacillus pentosus* GMNL-77 orally, it demonstrated profound local skin effects including reduced erythematous scaling lesions and downregulation of proinflammatory cytokines, including TNF-α, IL-6, and the interleukin-23 (IL-23)/IL-17A in the skin of imiquimod-treated mice [129].

These results suggest that microbial stimuli can reorganize inflammatory networks systemically and cutaneously. Notably, TLR-2 signalling on keratinocytes and immune cells is essential in psoriasis control: TLR-2 deficiency in imiquimod models exacerbated dermatitis via reduced Treg numbers and impaired IL-10 production, underscoring the protective role of TLR-2 and IL-10 in skin inflammation [110]. Additionally, IL-10 has been shown to directly suppress psoriatic inflammation, reinforcing its potential for targeted therapy [130].

In theory, a topical probiotic could engage TLR-2 on keratinocytes and resident immune cells, inducing IL-10 and Treg activation, as well as upregulating A20 (TNFAIP3) to inhibit NF-κB-mediated TNF-α/IL-23/IL-17 signalling. This axis may reduce keratinocyte hyperproliferation and inflammatory chemokine production, offering localized immunomodulation directly at psoriatic plaques.

However, probiotic immunomodulation may exert context-dependent effects. *Lactobacillus johnsonii* (La1), for example, administered orally, displayed a strong inhibition of UV-induced systemic IL-10 elevation [111]. By inhibiting overproduction of IL-10, the probiotic preserved essential cutaneous immune responses such as Langerhans cell population and contact hypersensitivity. These findings suggest that the immunomodulatory effects of probiotics are context-dependent and vary depending on the pathology. In chronic inflammatory diseases such as psoriasis and AD, increasing IL-10 expression is beneficial due to its anti-inflammatory and regulatory properties. Conversely, reducing IL-10 levels may be beneficial in situations of non-autoimmune tissue damage, where a stronger immune response is essential for repair and defence [111,126,127,129,130].

### 3.3. Competitive Inhibition and Biofilm Disruption

Probiotics outcompete pathogens via competitive exclusion, nutrient competition, and biofilm destabilization.

In a study by Chae et al. (2021) [112], two strains *of Lactiplantibacillus plantarum* isolated from green tea suppressed *S. aureus*, *Cutibacterium acnes*, and *Malassezia* spp. These antimicrobial activities were dose-dependent and accounted for by plantaricin-encoding genes, indicating a direct biochemical attack against pathogenic species via membrane disruption and nutrient competition [112]. This antimicrobial potential is further supported by findings from Tsai et al. (2021), who showed that *Lactiplantibacillus plantarum*-GMNL6 not only reduced biofilm formation by *S. aureus* but also inhibited the proliferation of *C. acnes*, demonstrating broad-spectrum pathogen control relevant to dermatological health [131].

Following these observations, Negi et al. (2025) explored deep-sea-derived probiotics—specifically *Lactococcus lactis* L25_4 and *Leuconostoc pseudomesenteroides* L25_6 and L25_7—and showed that these strains destabilized MRSA biofilms, downregulated bacterial gene expression related to membrane integrity, and upregulated the host AMP Cecropin in an ex vivo pork skin model and in vivo *Galleria mellonella* model, promoting host survival rates by over 90% [113].

A first-in-human study using live *Roseomonas mucosa* applied to patients with AD reported significant clinical improvements. Participants experienced reduced SCORAD scores and a lower need for corticosteroids. Moreover, *R. mucosa* was found not only to lower *S. aureus* levels but also to produce sphingolipids and reduce inflammation. This highlights the beneficial relationship between restoring the microbiome and modulating the immune system [114]. Additionally, a randomized clinical trial using autologous strains of *Staphylococcus hominis* and *Staphylococcus epidermidis* applied topically to replace *S. aureus* showed a significant reduction in pathogenic colonization and better clinical scores, with no adverse effects [115]. This demonstrates the safety and precision of these microbiome interventions.

These findings highlight the dual mechanism of probiotics: modulating host immunity while also directly suppressing pathogen virulence.

### 3.4. Barrier Restoration and Decreased Transepidermal Water Loss (TEWL)

Probiotic–epithelial interactions stimulate the expression of tight junction proteins and lipid-synthesizing enzymes, thereby strengthening the skin barrier and reducing transepidermal water loss (TEWL) [116,132,133].

In healthy volunteers after tape stripping, in reactive state, a *Bifidobacterium longum* lysate lowered TEWL significantly compared to placebo, indicating repair of the skin barrier. Ex vivo experiments on human skin confirmed reduced markers of inflammation such as vasodilation, oedema, and TNF-α release [117]. Additionally, a randomized double-blind self-control clinical trial evaluated a probiotic lotion containing fermented lysates of multiple probiotic strains of *Lactobacillus* spp. (*L. rhamnosus E06*, *L. paracasei E12*, *L. plantarum E15*, and *L. helveticus Y21*). There was a statistically significant decrease in the subjects’ TEWL (*p* < 0.01), with improved skin moisturization and reduced redness. This is direct evidence of the reinforcement of the barrier by topical treatment with probiotic laminate [116].

### 3.5. Probiotic Metabolite Production and Dermatological Impact

Current experimental studies highlight that topical probiotics produce a variety of bioactive metabolites (including organic acids, bacteriocins, and lipoteichoic acid) that contribute to the well-being of the skin. Probiotic effects are more extensive in biophysical modulation of the skin environment [112,121]. The generation of organic acids, lactic acid and acetic acid, by *Lactobacillus* spp., reduces local skin pH, rendering the environment less favourable for the proliferation of pathogens like *S. aureus* and *C. acnes* [106].

Lactic acid bacteria (LAB) have long been recognized for their beneficial effects on human health, including that of the skin. As outlined by Jeong et al. (2015), LAB like *L. plantarum* not only support the maintenance of skin barrier function but also exert anti-photoaging effects by modulating matrix metalloproteinase-1 (MMP-1) expression through their metabolite lipoteichoic acid [120]. This inhibition of collagen degradation encourages the preservation of skin elasticity and shape. In addition, LAB-derived metabolites also preserve the acidic pH of the skin, which is necessary for preventing pathogenic colonization and maintaining barrier function. Aside from transdermal benefits, oral intake of LAB has been shown to exert systemic immunomodulatory effects that indirectly encourage skin well-being by modulating gut-skin axis interaction [11,120,134].

Further, novel therapeutic modalities harness LAB’s ability to produce bioactive molecules directly at the wound site. For example, genetically modified *Limosilactobacillus reuteri* that secretes the chemokine CXCL12 is demonstrated to promote improved wound healing in minipig models by promoting the formation of granulation tissue and epithelial regeneration, underpinning the regenerative potential of probiotic metabolites in dermatology [135]. Furthermore, in wound-healing models, Li et al. (2025) [136] incorporated *L. plantarum*-derived metabolites into chitosan hydrogels. These secreted compounds—likely including short peptides and lipoteichoic acid—accelerated tissue regeneration, promoted epithelial proliferation, and reduced scarring, illustrating the wound-healing and regenerative potential of probiotic metabolites [120,136].

Topical application of *L. plantarum* in *Pseudomonas aeruginosa*-infected burns or chronic venous ulcers has shown immunomodulatory effects, including accelerating the healing process by inhibiting bacterial proliferation, accelerating tissue regeneration and modulating inflammation. The therapy decreased the number of neutrophils, apoptotic cells, and necrotic cells in the wound bed and regulated the production of IL-8, reducing increased levels provoked with *P. aeruginosa*, demonstrating its ability to control excessive inflammation and help tissue [137,138,139].

Aside from improving tissue healing in wounds, LAB-derived metabolites also play important roles in reducing environmental damage. These multifunctional bioactive compounds could hold wider significance in dermatology, especially during ultraviolet (UV) exposure [119,120]. In UVB-exposed human keratinocyte (HaCaT) cells and hairless mice, Park et al. (2024) demonstrated that *Limosilactobacillus fermentum* MG5368 and *Lactiplantibacillus plantarum* MG989 secreted lactic acid and other organic acids, which reduced UVB-induced oxidative stress, suppressed inflammatory mediators (e.g., MMPs), and enhanced collagen synthesis [118]. These acidic metabolites not only regulate local pH but also support antioxidant defences and barrier repair [119,120].

Overall, these findings underline the multifunctional roles of probiotic strains and their metabolites in promoting skin integrity, immune modulation, and tissue repair.

Table 5 summarizes selected LAB metabolites with reported dermatological activity, including their producing strains, biological activities, and experimental contexts. The table highlights how specific compounds, including lipoteichoic acid, organic acids, plantaricins, and exopolysaccharides, have antimicrobial, antioxidant, anti-inflammatory, and barrier-improving effects in both in vitro and in vivo models. This variety of action shows the therapeutic flexibility of LAB in dermatological uses, from UV protection to pathogen inhibition and skin barrier support.

### 3.6. Systemic Immunomodulation via the Gut–Skin Axis: The Role of Oral Probiotics

Aside from local (topical) application, oral probiotic therapy has been shown to produce systemic effects through the gut–skin axis, a bidirectional communication network between gastrointestinal and cutaneous immune systems.

Qi et al. (2024) [144] conducted an in vivo study using an LL-37-induced rosacea-like mouse model and demonstrated that oral intake using *Ligilactobacillus* spp. successfully alleviated inflammation. These actions were via the downregulation of TLR2, MyD88, and NF-κB, resulting in the decreased expression of LL-37 and pro-inflammatory cytokines such as TNF-α, interleukin-1-beta (IL-1β), and IL-6 [144]. Similarly, oral *Lactobacillus acidophilus* enhanced the healing of wounds by promoting collagen synthesis and epithelialization, reporting the systemic, immunomodulatory, and regenerative effects induced by the gut-skin axis. Compared with *L. plantarum*, *L. acidophilus* was seen to relate to more wound contraction, earlier epithelialization, higher breaking strength of the wound, and higher collagen level in granulation tissue. These results indicate that LAB strains may have varying positive effects depending on the nature of the strain and the disease being treated [133].

Oral probiotics such as *Bifidobacterium infantis* and *Lactobacillus pentosus* were discovered in AD and psoriasis to rebalance Th17/Treg responses, reduce systemic levels of TNF-α and IL-6, and reduce disease severity, suggesting that gut microbial modulation can repress chronic skin inflammation and ensure cutaneous immune tolerance [129,145,146].

**Figure 5:**
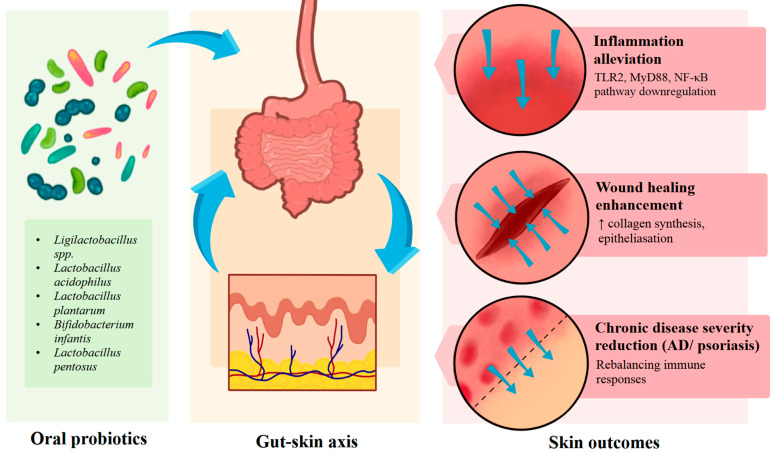
Oral probiotics action through the gut–skin axis: a summary. Abbreviations: TLR2—Toll-like receptor 2; MyD88—Myeloid differentiation primary response 88; NF-κB—Nuclear factor kappa-light-chain-enhancer of activated B cells; AD—atopic dermatitis.

Figure 5 summarizes the above-discussed effects produced through the gut–skin axis by oral probiotic agents.

The table below (Table 6) summarizes key microbiome-related alterations implicated in the pathogenesis of major skin diseases and outlines how topical probiotics counteract these mechanisms. Highlighted are their immunologic and antimicrobial actions, as well as their observed or potential effects on disease progression and treatment outcomes.

**Figure 6:**
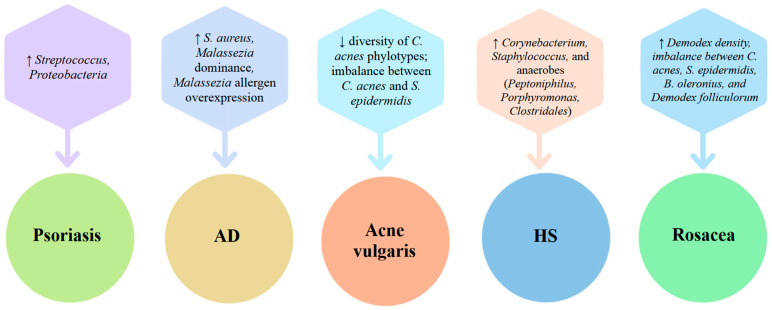
Microbial factors contributing to the development of dermatologic conditions. Abbreviations: AD—atopic dermatitis; HS—Hidradenitis suppurativa.

Figure 6 illustrates which microorganisms influence the development of skin diseases, such as psoriasis, AD, acne vulgaris, HS, and rosacea.

## 4. Materials and Methods

This narrative review was based on a comprehensive literature search across PubMed, Scopus, and Google Scholar databases, encompassing both clinical trials and experimental research in animal models. The primary focus was on publications from the past decade, although relevant earlier landmark studies from the broader period 1975–2025 were also incorporated. The literature search employed combinations of Medical Subject Headings (MeSH) and free-text keywords covering topics with time filters applied to identify the most relevant studies. Core search terms included combinations of: “skin microbiota”, “dysbiosis”, “probiotics”, “topical probiotics”, “inflammatory dermatoses”, “atopic dermatitis”, “psoriasis”, and “acne”. Figure 1, Figure 2, Figure 3 and Figure 4 included in this article were created by the authors. Each figure was developed based on a synthesis of the reviewed literature to accurately reflect current evidence and enhance the clarity of the presented concepts.

## 5. Conclusions

The microbiota plays a significant role in maintaining proper skin function. Disturbances in microbial balance are not merely a reflection of skin pathology but also constitute an integral part of the pathogenesis and contribute to the development of skin conditions, including atopic dermatitis, psoriasis, acne vulgaris, hidradenitis suppurativa, rosacea, impaired wound healing, and photoaging. Topical probiotics, whether through live microorganisms, their lysates or secreted metabolites, represent a promising therapeutic approach for chronic inflammatory dermatoses linked to skin microbiota dysbiosis, with current evidence indicating their potential to modulate local immune responses. The primary effects of topical probiotic application include restoration of microbiome balance, reinforcement of skin barrier function, and suppression of pathogenic and opportunistic microorganisms implicated in inflammatory dermatoses. The key mechanisms underlying these effects are production of antimicrobial peptides like bacteriocins, modulation of cytokine expression, and enhancement of tight junction proteins. Clinical studies, although limited by small sample sizes and heterogeneous methodologies, consistently demonstrate improvements in clinical outcomes, enhancement of cutaneous defence mechanisms, and reduced colonization by pathogenic species. Additional benefits, supported by both clinical observations and experimental studies, include accelerated skin regeneration—such as improved wound healing—and mitigation of environmental stressors, including UV-induced damage.

Despite growing scientific interest, this approach is still insufficiently studied and requires further clinical investigation. Overall, evidence from the literature suggests that the key research challenges associated with topical probiotics usage include the lack of standardization in material collection, dosing, and the selection scheme of species and strains contained in the preparation. Another major challenge posed is the lack of data on the long-term effects of topical probiotic use on health—especially in paediatric patients or those under immunosuppression.

Translating the promise of topical probiotics into clinical practice will require not only rigorous clinical validation but also the establishment of regulatory standards ensuring quality, safety, and reproducibility. Progress in this field will depend on close interdisciplinary collaboration among dermatologists, microbiologists, immunologists, and formulation scientists to optimize strain selection, delivery systems, and patient-tailored interventions.

## Figures and Tables

**Table 1:** Topographical Distribution of Skin Microbiota Across Distinct Cutaneous Microenvironments [3,6,19,23,24,25].

Skin Site Type	Physiological Features	Representative Anatomical Locations	Dominant Bacterial Taxa
Sebaceous Sites [3,6,23,24]	Low moisture, high lipid content; acidic due to free fatty acids	Glabella, alar crease, external auditory canal, back, upper chest, face	*Cutibacterium* (*Propionibacteriaceae*), *Staphylococcaceae*, *Corynebacteriaceae*
		Occiput	*Staphylococcaceae*, *Corynebacteriaceae*, *Proteobacteria*
Moist Sites [3,6,25]	High humidity and temperature; presence of glands and folds	Axillary vault, antecubital fossa, popliteal fossa, plantar heel	*Proteobacteria*, *Staphylococcaceae*, *Bacteroidetes*
		Inguinal crease, umbilicus, gluteal crease	*Corynebacteriaceae*, *Staphylococcaceae*
		Umbilicus	*Corynebacteriaceae*
		Toe web space	*Corynebacteriaceae*, *Staphylococcaceae*, *Cyanobacteria*
Dry Sites [3,6,19]	Lower humidity; high microbial diversity but low temporal stability	Volar forearm, hypothenar palm, interdigital web space, plantar heel	*Proteobacteria*, *Streptococcaceae*, *Actinobacteria* (*various*), *Bacteroidetes*

**Table 2:** Cytokine and T-Cell Responses Triggered by Skin Commensals [46].

Commensal Species	Cytokine Induction	Immune Cell Activation & Localization
*Staphylococcus epidermidis*	↑ IL-17A	Induces Th17 and CD8+ T cells localized in the epidermis; CD8+ T cells produce IL-17A/IFN-γ and enhance barrier immunity
*Cutibacterium acnes*, *Staphylococcus aureus*	↑ IL-17A, ↑ IFN-γ	Expand skin-resident IL-17A+ and IFN-γ T cells, but no CD8+ T cells response comparable to *Staphylococcus epidermidis*

Abbreviations: IL—Interleukin 17; IFN-γ—Interferon gamma; Th17—T helper 17 cells; CD8+ T cells—CD8+ T lymphocytes.

**Table 3:** Summary of skin microbiome alterations in AD [58,70,73,81,82,83].

Author (Year)	Study Group	Sample Type	Origin of Skin Samples	Bacteria/Fungi Alterations in AD
Zhang et al., 2011 (study about skin fungal microbiota) [73]	9 patients with AD (3 each with mild, moderate and severe disease)	Scale samples collected using 7 cm × 9 cm OpSite strips (Smith & Nephew, Hull, UK) (Sugita method); each site sampled three times.	Scale samples collected from facial lesional sites (patients) and non-lesional skin (controls)	*Malassezia*—Mild/moderate AD: *M. restricta* > *M. globosa.* Severe AD: ratio *M. restricta*: *M. globosa* ≈ 1. Non-*Malassezia* yeasts: more diverse in AD (13.0 ± 3.0 spp.) vs. healthy (8.0 ± 1.9 spp.).
Fyhrquist et al., 2019 [58]	AD (*n* = 91); Controls (*n* = 126)	Skin samples collected using a sterile 2.5 cm ring filled with 1.5 mL PBS; skin scraped with glass rod (10× left, 10× left), no prior cleaning.	Skin samples collected from upper/lower back, posterior thigh, or buttocks	↑ *S. aureus* (not in all lesions → possible endotypes) Loss of anaerobes (e.g., *Lactobacillus*, *Finegoldia*) → switch to aerobic metabolism *S. aureus* negatively correlates with *S. epidermidis* and *Corynebacterium* spp.
Edslev et al., 2021 (Staphylococcus comparison) [70]	AD (*n* = 94); Controls (*n* = 92)	Skin swab prepared using eSwabs (Copan, Brescia, Italy)	AD: lesional and non-lesional samples collected from the volar forearm and the cubital crease; Control: the antecubital crease	Severity of AD was associated with alterations in the *Staphylococcus* community. ↑ *S. aureus*, *S. capitis*, *S. lugdunensis* → directly correlated with disease severity. ↓ *S. hominis* → inversely correlated with disease severity; reduced abundance compared with healthy skin.
Suwarsa et al., 2021 [81]	AD (*n* = 12), 9 with mild disease and 3 moderate disease; Controls (*n* = 4)	Skin swab, sterile, pre-moistened swab rubbed for 20 s	Samples collected from volar forearm (cubital fossa)	Moderate AD—Dominance of *Firmicutes*, *Bacilli*, *Bacillales Staphylococcaceae*, *Staphylococcus*; highest abundance of *S. aureus*; reduced microbial diversity. Mild AD—Dominance of *Proteobacteria*, *Gammaproteobacteria*, *Pseudomonadales*, *Moraxellaceae*, *Acinetobacter*.
Schmid et al., 2022 [82]	AD (*n* = 16); Controls (*n* = 16)	Skin swab—flocked swabs (Floqswabs/eSwabs, COPAN, Brescia, Italy) pre-soaked in 0.9% NaCl (0.9%, Braun, Sempach, Switzerland); rubbed repeatedly over 4–8 cm^2^ of skin.	Skin swabs collected from antecubital crease, dorsal neck, glabella and vertex	↑ *S. aureus*, ↓ *Cutibacterium* spp. Severe AD: *Malassezia* predominant, but ↑ non-*Malassezia* fungi (e.g., *Candida*, *Debaryomyces*); ↓ *M. restricta*, ↓ *M. sympodialis*, ↑ *M. furfur* compared to healthy individuals and mild-to-moderate AD.
Kim et al., 2024 [83]	AD (*n* = 20); Controls (*n* = 16)	Skin samples—swabs (TransportsystemTM 108C; Copan Diagnostics Inc., Murrieta, CA, USA) and tape strips (Cuderm Corporation, Dallas, TX, USA)	AD: lesional scalp and non-lesional scalp (at least 4 cm from the lesional skin)	↑ *Staphylococcus* spp. and *Kocuria* spp. ↓ *Cutibacterium* and *Lawsonella*

Abbreviations: AD—atopic dermatitis; PBS—phosphate-buffered saline; NaCl—sodium chloride; CA—California; USA—United States of America.

**Table 4:** Summary of Microbiome Alterations in Psoriatic Skin Based on 16S rRNA Sequencing [86,93,94,95,96,97,98,99,100,101].

Author (Year)	Study Group	Sample Type	Origin of Skin Samples	Bacteria Increased	Bacteria Decreased
Gao et al., 2008 [93]	Psoriasis patients (*n* = 6)	Skin biopsy: - Unaffected skin: 1 sample - Psoriatic lesions: ≥2 samples	Forearm/finger/elbow/shoulder/back/abdomen/leg/knee/arm	*Firmicutes*, *Streptococcus*	*Actinobacteria*, *Propionibacterium*, *Proteobacteria*
Fahlen et al., 2012 [94]	Psoriasis (*n* = 10), Controls (*n* = 12)	Skin biopsy: - Psoriasis: 2 mm biopsies from plaques - Control: 2 × 2 mm biopsies from excised lesions	Psoriasis: 4 trunk (3 back, 1 flank), 6 limbs (3 arm, 3 leg)Control: 8 trunk (6 back, 1 abdomen, 1 chest), 4 limbs (3 arms, 1 leg), 1 neck	*Proteobacteria*, *Streptococcus*	*Propionibacteria*, *Staphylococcus*
Alekseyenko et al., 2013 [95]	Psoriasis (*n* = 54), Controls (*n* = 37)	Skin swab (2 × 2 cm area, cotton pledget soaked in 0.15 M NaCl + 0.1% Tween 20): - Psoriasis: Lesion (plaque) and unaffected (contralateral) - Control	Psoriasis: face/scalp/back/abdomen/shoulder/arm/elbow/forearm/leg/thigh/knee/shin/foot/Control: 4 standardized sites per person (scalp, abdomen, inner elbow, kneecap)	*Corynebacterium*, *Propionibacterium*, *Staphylococcus*, *Streptococcus*	*Cupriavidus*, *Flavisolibacter*, *Methylobacterium*, *Schlegelella*
Drago et al., 2016 [96]	Psoriasis (*n* = 1), Controls (*n* = 1)	Skin biopsy (2 cm^2^ via curettage): - Psoriasis: 2 lesional and 2 non-lesional samples - Control: 2 samples	Psoriasis and control: the area behind the left ear	*Proteobacteria*, *Bacteroidetes*, *Streptococcus*, *Rhodobacteraceae*, *Campylobacteraceae*, and *Moraxellaceae*	*Staphylococcus*, *Propionibacteriaceae*
Tett et al., 2017 [97]	Psoriasis (*n* = 28)	Skin swab (based on the protocol validated and adopted by the HMPC, sterile cotton-tipped swabs (VWR, Milan, Italy) were moistened with SCF-1 buffer *	Psoriasis: the olecranon skin area and the retroauricular crease (behind the ear) from left and left body site	*Staphylococcus*, Novel/uncultured taxa (*Anaerococcus* spp., related *Chromobacteriaceae/Neisseriaceae*, novel *Malassezia*)	Overall microbial diversity ↓
Chang et al., 2018 [98]	Psoriasis (*n* = 28), Controls (*n* = 26)	Skin swab (individually packed, sterile Epicentre Catch-All swabs): - Psoriasis: lesional + non-lesional samples - Control	Control and psoriasis non-lesional: 6 standardized sites per person (scalp, trunk, axilla, arm, leg, gluteal fold) Psoriasis lesional: only from sites with visible plaques among the 6	*Staphylococcus aureus*, *Proteobacteria*	*Staphylococcus epidermidis*, *Cutibacterium acnes*, *Actinobacter*
Assarsson et al., 2018 [99]	Psoriasis (*n* = 26)	Skin swab (4 × 4 cm area, flocked swab soaked in 1 mL liquid Amies [ESwab™, Copan, Brescia, Italy]): Psoriasis - Lesional: target plaque - Non-lesional: adjacent site ≥ 10 cm from lesion	All samples from dry micro-environments	Firmicutes	*Staphylococcus*
Stehlikova et al., 2019 [100]	Psoriasis (*n* = 34), Controls (*n* = 25)	Skin swab (2 × 2 cm, FLOQSwabs™ COPAN Diagnostics Inc., United States, SCF-1 buffer *); Skin scraping (2 × 2 cm, scalpel, SCF-1 buffer *); Skin biopsy (2 mm punch, dry stored);—Psoriasis: lesional + non-lesional samples - Control	Control and psoriasis non-lesional: samples from dorsal (back) or olecranon (elbow) skin areas	*Brevibacterium*, *Kocuria palustris*, *Gordonia*	*Staphylococcus*; *Propionibacterium* compared to healthy skin on elbow
Assarsson et al., 2020 [86]	Psoriasis (*n* = 39), Controls (*n* = 70)	Skin swab (4 × 4 cm; using a flocked swab pre-moistened with 1 mL of liquid Amies medium (ESwab™, Copan Diagnostics Inc., Murrieta, CA, USA)	Control: pharynx and elbow skin; Psorasis: pharynx, lesional skin of elbow, adjacent non-lesional skin (≥10 cm from lesion)	*Corynebacterium*, 4 genera correlated with severity—*Capnocytophaga*, *Leptotrichia*, *Abiotrophia* and *Tanne-rella*	*Streptococcus gordonii*, *Cutibacterium*, *Prevotella*
Kayıran et al., 2022 [101]	Psoriasis (*n* = 10), Controls (*n* = 10)	Skin swab (rubbing swabs soaked sterile in DNA/RNA Shield™, Zymo Research, Irvine, CA, USA	Control: scalp Psoriasis: lesional and non-lesional hairy scalp	*Staphylococcus*, *Streptococcus*, *Aquabacterium*, *Neisseria*, *Azospirillum*, *Mycobacterium*, *Finegoldia*, *Haemophilus*, *Ezakiella*	*Propionibacterium*

* SCF-1 buffer (50 mM Tris buffer, 1 mM EDTA, 0.5% Tween 20; HMPC); Abbreviations: NaCl—Sodium chloride; EDTA—Ethylenediaminetetraacetic acid; HMPC—Human Microbiome Project Consortium; CA—California; DNA—Deoxyribonucleic acid; RNA—Ribonucleic acid.

**Table 5:** Selected LAB Metabolites with Demonstrated Dermatological Effects [112,118,128,131,140,141,142,143].

Metabolite Class	Producing Strain Examples	Mechanisms of Action and Demonstrated Effects	Experimental Model In Vitro	Experimental Model In Vivo	Reference Number
LTA	*Lactobacillus plantarum* K8	Inhibits MMP-1, suppresses ERK/JNK/AP-1/NF-κB, reduces ROS, increases type I procollagen	UVB-irradiated human dermal fibroblasts	-	[140]
Organic acids (lactic, acetic)	*Lactobacillus plantarum*, *L. fermentum*	Lowers skin pH, inhibits *S. aureus* and *C. acnes*, reduces oxidative stress, suppresses inflammatory mediators, promotes barrier function	HaCaT keratinocytes	UVB-stressed mouse skin	[118]
Plantaricins (bacteriocins)	*Lactiplantibacillus plantarum* (APsulloc 331261/266)	Suppresses *S. aureus*, *C. acnes*, *Malassezia* spp.; inhibits biofilm formation, destabilizes membrane integrity	Agar diffusion, co-culture, gene profiling	-	[112]
Peptidoglycan fragments	*L. plantarum*-GMNL6	Stimulates collagen synthesis, upregulates SPTSSA, inhibits *C. acnes* and *S. aureus* biofilms	Skin models	observational clinical data in humans	[131]
Lipoteichoic acid & SCFAs	*L. plantarum*, *L. casei*	Enhance tight junction proteins, reduce TEWL, modulate cutaneous immune signaling via TLR2/NF-κB	Keratinocyte models	mouse skin assays	[128]
EPS	*Lactobacillus casei*, *L. rhamnosus*	Antioxidant, improves moisture retention, enhances barrier regeneration	Topical gel formulations	clinical skin hydration evaluations	[141,142]
Biosurfactants	*L. plantarum*, *L. jensenii*	Prevent adhesion of *S. aureus*, reduce biofilm persistence, modulate surface tension	Surface adhesion tests, microplate assays	-	[143]

Abbreviations: LTA—Lipoteichoic acid; MMP-1—Matrix metallopeptidase 1; ERK—Extracellular signal-regulated kinase; JNK—c-Jun N-terminal kinase; AP-1—Activator protein-1; NF-κB—Nuclear factor kappa-light-chain-enhancer of activated B cells; ROS—Reactive oxygen species; SCFAs—Short-chain fatty acids; SPTSSA—Serine palmitoyltransferase small subunit A; EPS—Exopolysaccharides; TEWL—Transepidermal water loss; TLR2—Toll-like receptor 2; HaCaT—Human keratinocyte cell line; UVB—Ultraviolet B radiation.

**Table 6:** Microbiome-Targeted Effects of Topical Probiotics Across Dermatologic Conditions [4,5,57,58,72,73,74,75,86,90,93,94,95,96,98,99,104,114,115,118,119,120,125,126,127,136,137,138,139,144,147,148,149,150,151,152,153,154,155,156,157,158,159,160,161].

Disease/Indication	Key Microbiome Alterations in Pathogenesis	Key Mechanism(s) of Topical Probiotics	Effects on Disease Course/Treatment
Psoriasis	↓ microbial diversity; ↑ *Streptococcus* and other *Firmicutes*; variable *Proteobacteria* enrichment (strain-level differences); ↓ commensal *Actinobacteria* (*Cutibacterium*, *S. epidermidis*) * [58,86,90,93,94,95,96,98,99,104]	Immune modulation: dampening IL-1β/TNFα cascade; restoring barrier-microbiome balance; inhibition and enhancement of gene expression [147,148]	Reduced lesion inflammation; reduced TEWL, improved barrier; potentially decreased need for topical steroids [148,149]
Atopic Dermatitis (AD)	↓ microbial diversity; overgrowth of *S. aureus* and opportunistic *Staphylococci*; suppression of *S. epidermidis* and *Corynebacterium*; fungal dysbiosis (*Malassezia* dominance, Malassezia allergen overexpression) [5,57,58,72,73,74,75]	Recolonization with commensals (e.g., *S. epidermidis*, *Vitreoscilla filiformis*, *Roseomonas mucosa*): *S. aureus* inhibition; upregulates AMPs (cathelicidin); modulates TLR2-mediated innate immune responses; reduces integrin-mediated infiltration, increases TNFAIP3/A20 expression [126,127].	Improvement in eczema severity; decreased *S. aureus* colonization; itch reduction and barrier restoration, enhancement in cutaneous homeostasis [114,115]
Acne vulgaris	↓ microbial diversity; ↓ diversity of C. acnes phylotypes; imbalance between *C. acnes* and *S. epidermidis* [4,155]	Strain-specific inhibition of *C. acnes* by succinic acid/fermentation by *S. epidermidis* or *L. plantarum*; anti-inflammatory IL-8 modulation; boosting AMP expression, destabilizing MRSA biofilms [125,150].	Reduction in pustules/inflammatory lesions; reduced bacterial load; diminished IL-8 and cytokine-driven inflammation [150,151,152]
Hidradenitis suppurativa (HS)	Dysbiosis with overgrowth of *Corynebacterium*, *Staphylococcus*, and anaerobes (*Peptoniphilus*, *Porphyromonas*, *Clostridales*);↓ commensals (e.g., *Cutibacterium)*;↓ niche heterogeneity [161]	Competitive exclusion of pathogenic flora; SCFA-mediated suppression of inflammation; AMP induction [153,154]	Potential reduction in abscess formation and inflammation; microbiome normalization; improved wound healing [153,154]
Rosacea	Increased *Demodex* density; ↑ TLR2 overexpression; ↑ AMPs (e.g., cathelicidins); dysbiosis of cutaneus microbiota (imbalance between *C. acnes*, *S. epidermidis, B. oleronius*, and *Demodex folliculorum*) [156,157]	Downregulation of TLR2–NF-κB–IL-8 pathway; reduction in LL-37 and ROS [144]	Reduced erythema, papules, and sensitivity; restoration of immune balance and microbial diversity [144,149]
Photoaging/Skin Aging	UV exposure alters skin microbiome diversity and metabolic capacity; decreased antioxidant pathways, increased MMP activity [159,160]	Reduction in oxidative stress, inhibition of MMP and AP-1/NF-κB pathways; transcriptional suppression, immune homeostasis and collagen synthesis support [118].	Reduced wrinkle formation, improved elasticity and hydration; prevention of UV-induced ECM degradation [119,120].

* Inter-study variability. Abbreviations: IL—Interleukin; TNFα—Tumor necrosis factor alpha; TEWL—Transepidermal water loss; AMPs—Antimicrobial peptides; MRSA—Methicillin-resistant *Staphylococcus aureus*; TLR2—Toll-like receptor 2; SCFA—Short-chain fatty acid; NF-κB—Nuclear factor kappa-light-chain-enhancer of activated B cells; MMP—Matrix metallopeptidase; AP-1—Activator protein-1; UV—Ultraviolet radiation; ECM—Extracellular matrix; ROS—Reactive oxygen species.

## Data Availability

No new data were created or analyzed in this study. Data sharing is not applicable to this article.

## References

[1] Gilchrest B.A. (2003). Skin Aging 2003: Recent Advances and Current Concepts. Cutis.

[2] Chung J.H., Seo J.Y., Choi H.R., Lee M.K., Youn C.S., Rhie G., Cho K.H., Kim K.H., Park K.C., Eun H.C. (2001). Modulation of Skin Collagen Metabolism in Aged and Photoaged Human Skin In Vivo. J. Investig. Dermatol..

[3] Grice E.A., Segre J.A. (2011). The Skin Microbiome. Nat. Rev. Microbiol..

[4] Dréno B., Dagnelie M.A., Khammari A., Corvec S. (2020). The Skin Microbiome: A New Actor in Inflammatory Acne. Am. J. Clin. Dermatol..

[5] Kong H.H., Oh J., Deming C., Conlan S., Grice E.A., Beatson M.A., Nomicos E., Polley E.C., Komarow H.D., Murray P.R. (2012). Temporal Shifts in the Skin Microbiome Associated with Disease Flares and Treatment in Children with Atopic Dermatitis. Genome Res..

[6] Grice E.A., Kong H.H., Conlan S., Deming C.B., Davis J., Young A.C., Bouffard G.G., Blakesley R.W., Murray P.R., Green E.D. (2009). Topographical and Temporal Diversity of the Human Skin Microbiome. Science.

[7] Šuler Baglama Š., Trčko K. (2022). Skin and Gut Microbiota Dysbiosis in Autoimmune and Inflammatory Skin Diseases. Acta Dermatovenerol. Alp. Pannonica Adriat..

[8] Borrego-Ruiz A., Borrego J.J. (2024). Nutritional and Microbial Strategies for Treating Acne, Alopecia, and Atopic Dermatitis. Nutrients.

[9] Rozas M., Hart de Ruijter A., Fabrega M.J., Zorgani A., Guell M., Paetzold B., Brillet F. (2021). From Dysbiosis to Healthy Skin: Major Contributions of *Cutibacterium acnes* to Skin Homeostasis. Microorganisms.

[10] De Almeida C.V., Antiga E., Lulli M. (2023). Oral and Topical Probiotics and Postbiotics in Skincare and Dermatological Therapy: A Concise Review. Microorganisms.

[11] da Silva Vale A., de Melo Pereira G.V., de Oliveira A.C., de Carvalho Neto D.P., Herrmann L.W., Karp S.G., Soccol V.T., Soccol C.R. (2023). Production, Formulation, and Application of Postbiotics in the Treatment of Skin Conditions. Fermentation.

[12] Gallo R.L. (2017). Human Skin Is the Largest Epithelial Surface for Interaction with Microbes. J. Investig. Dermatol..

[13] Cundell A.M. (2018). Microbial Ecology of the Human Skin. Microb. Ecol..

[14] Murillo N., Raoult D. (2013). Skin Microbiota: Overview and Role in the Skin Diseases Acne Vulgaris and Rosacea. Future Microbiol..

[15] Andersen B.M. (2019). Prevention and Control of Infections in Hospitals.

[16] Byrd A.L., Belkaid Y., Segre J.A. (2018). The Human Skin Microbiome. Nat. Rev. Microbiol..

[17] Hannigan G.D., Meisel J.S., Tyldsley A.S., Zheng Q., Hodkinson B.P., SanMiguel A.J., Minot S., Bushman F.D., Grice E.A. (2015). The Human Skin Double-Stranded DNA Virome: Topographical and Temporal Diversity, Genetic Enrichment, and Dynamic Associations with the Host Microbiome. mBio.

[18] Jacob S., VanDaele M.A., Brown J.N. (2019). Treatment of *Demodex*-associated Inflammatory Skin Conditions: A Systematic Review. Dermatol. Ther..

[19] Oh J., Byrd A.L., Park M., Kong H.H., Segre J.A. (2016). Temporal Stability of the Human Skin Microbiome. Cell.

[20] Murillo N., Aubert J., Raoult D. (2014). Microbiota of Demodex Mites from Rosacea Patients and Controls. Microb. Pathog..

[21] Kloos W.E., Musselwhite M.S. (1975). Distribution and Persistence of *Staphylococcus* and *Micrococcus* Species and Other Aerobic Bacteria on Human Skin. Appl. Microbiol..

[22] Noble S.M., Gianetti B.A., Witchley J.N. (2017). Candida Albicans Cell-Type Switching and Functional Plasticity in the Mammalian Host. Nat. Rev. Microbiol..

[23] Somerville D.A. (1969). The normal flora of the skin in different age groups. Br. J. Dermatol..

[24] Fluhr J.W., Kao J., Ahn S.K., Feingold K.R., Elias P.M., Jain M. (2001). Generation of Free Fatty Acids from Phospholipids Regulates Stratum Corneum Acidification and Integrity. J. Investig. Dermatol..

[25] Roth R.R., James W.D. (1988). Microbial Ecology of the Skin. Annu. Rev. Microbiol..

[26] Kong H.H. (2011). Skin Microbiome: Genomics-Based Insights into the Diversity and Role of Skin Microbes. Trends Mol. Med..

[27] Ursell L.K., Clemente J.C., Rideout J.R., Gevers D., Caporaso J.G., Knight R. (2012). The Interpersonal and Intrapersonal Diversity of Human-Associated Microbiota in Key Body Sites. J. Allergy Clin. Immunol..

[28] McLoughlin I.J., Wright E.M., Tagg J.R., Jain R., Hale J.D.F. (2022). Skin Microbiome—The Next Frontier for Probiotic Intervention. Probiotics Antimicrob. Proteins.

[29] Meisel J.S., Sfyroera G., Bartow-McKenney C., Gimblet C., Bugayev J., Horwinski J., Kim B., Brestoff J.R., Tyldsley A.S., Zheng Q. (2018). Commensal Microbiota Modulate Gene Expression in the Skin. Microbiome.

[30] Williams M.R., Costa S.K., Zaramela L.S., Khalil S., Todd D.A., Winter H.L., Sanford J.A., O’Neill A.M., Liggins M.C., Nakatsuji T. (2019). Quorum Sensing between Bacterial Species on the Skin Protects against Epidermal Injury in Atopic Dermatitis. Sci. Transl. Med..

[31] Baldwin H.E., Bhatia N.D., Friedman A., Eng R.M., Seite S. (2017). The Role of Cutaneous Microbiota Harmony in Maintaining a Functional Skin Barrier. J. Drugs Dermatol..

[32] Pausan M.R., Csorba C., Singer G., Till H., Schöpf V., Santigli E., Klug B., Högenauer C., Blohs M., Moissl-Eichinger C. (2019). Exploring the Archaeome: Detection of Archaeal Signatures in the Human Body. Front. Microbiol..

[33] Grice E.A., Kong H.H., Renaud G., Young A.C., Bouffard G.G., Blakesley R.W., Wolfsberg T.G., Turner M.L., Segre J.A. (2008). A Diversity Profile of the Human Skin Microbiota. Genome Res..

[34] Kong H.H., Andersson B., Clavel T., Common J.E., Jackson S.A., Olson N.D., Segre J.A., Traidl-Hoffmann C. (2017). Performing Skin Microbiome Research: A Method to the Madness. J. Investig. Dermatol..

[35] Nakatsuji T., Chen T.H., Narala S., Chun K.A., Two A.M., Yun T., Shafiq F., Kotol P.F., Bouslimani A., Melnik A.V. (2017). Antimicrobials from Human Skin Commensal Bacteria Protect against *Staphylococcus aureus* and Are Deficient in Atopic Dermatitis. Sci. Transl. Med..

[36] O’Sullivan J.N., Rea M.C., O’Connor P.M., Hill C., Ross R.P. (2019). Human Skin Microbiota Is a Rich Source of Bacteriocin-Producing Staphylococci That Kill Human Pathogens. FEMS Microbiol. Ecol..

[37] Gaitanis G., Tsiouri G., Spyridonos P., Stefos T., Stamatas G.N., Velegraki A., Bassukas I.D. (2019). Variation of Cultured Skin Microbiota in Mothers and Their Infants during the First Year Postpartum. Pediatr. Dermatol..

[38] Sanford J.A., Gallo R.L. (2013). Functions of the Skin Microbiota in Health and Disease. Semin. Immunol..

[39] Maheswary T., Nurul A.A., Fauzi M.B. (2021). The Insights of Microbes’ Roles in Wound Healing: A Comprehensive Review. Pharmaceutics.

[40] Wanke I., Steffen H., Christ C., Krismer B., Götz F., Peschel A., Schaller M., Schittek B. (2011). Skin Commensals Amplify the Innate Immune Response to Pathogens by Activation of Distinct Signaling Pathways. J. Investig. Dermatol..

[41] Dréno B., Araviiskaia E., Berardesca E., Gontijo G., Sanchez Viera M., Xiang L.F., Martin R., Bieber T. (2016). Microbiome in Healthy Skin, Update for Dermatologists. J. Eur. Acad. Dermatol. Venereol..

[42] Friedrich A., Paz M., Leoni J., González Maglio D. (2017). Message in a Bottle: Dialog between Intestine and Skin Modulated by Probiotics. Int. J. Mol. Sci..

[43] Park Y.J., Lee H.K. (2018). The Role of Skin and Orogenital Microbiota in Protective Immunity and Chronic Immune-Mediated Inflammatory Disease. Front. Immunol..

[44] Coates M., Blanchard S., MacLeod A.S. (2018). Innate Antimicrobial Immunity in the Skin: A Protective Barrier against Bacteria, Viruses, and Fungi. PLoS Pathog..

[45] Naik S., Bouladoux N., Wilhelm C., Molloy M.J., Salcedo R., Kastenmuller W., Deming C., Quinones M., Koo L., Conlan S. (2012). Compartmentalized Control of Skin Immunity by Resident Commensals. Science.

[46] Naik S., Bouladoux N., Linehan J.L., Han S.-J., Harrison O.J., Wilhelm C., Conlan S., Himmelfarb S., Byrd A.L., Deming C. (2015). Commensal–Dendritic-Cell Interaction Specifies a Unique Protective Skin Immune Signature. Nature.

[47] Canesso M.C.C., Vieira A.T., Castro T.B.R., Schirmer B.G.A., Cisalpino D., Martins F.S., Rachid M.A., Nicoli J.R., Teixeira M.M., Barcelos L.S. (2014). Skin Wound Healing Is Accelerated and Scarless in the Absence of Commensal Microbiota. J. Immunol..

[48] Linehan J.L., Harrison O.J., Han S.-J., Byrd A.L., Vujkovic-Cvijin I., Villarino A.V., Sen S.K., Shaik J., Smelkinson M., Tamoutounour S. (2018). Non-Classical Immunity Controls Microbiota Impact on Skin Immunity and Tissue Repair. Cell.

[49] Boxberger M., Cenizo V., Cassir N., La Scola B. (2021). Challenges in Exploring and Manipulating the Human Skin Microbiome. Microbiome.

[50] Li H., Zhang Z., Zhang H., Guo Y., Yao Z. (2021). Update on the Pathogenesis and Therapy of Atopic Dermatitis. Clin. Rev. Allergy Immunol..

[51] Garmhausen D., Hagemann T., Bieber T., Dimitriou I., Fimmers R., Diepgen T., Novak N. (2013). Characterization of Different Courses of Atopic Dermatitis in Adolescent and Adult Patients. Allergy.

[52] Otto M. (2014). *Staphylococcus aureus* Toxins. Curr. Opin. Microbiol..

[53] Cheung G.Y.C., Joo H.-S., Chatterjee S.S., Otto M. (2014). Phenol-Soluble Modulins—Critical Determinants of Staphylococcal Virulence. FEMS Microbiol. Rev..

[54] Otto M. (2010). *Staphylococcus* Colonization of the Skin and Antimicrobial Peptides. Expert. Rev. Dermatol..

[55] Novick R.P. (2003). Autoinduction and Signal Transduction in the Regulation of Staphylococcal Virulence. Mol. Microbiol..

[56] Godakova S.A., Noskov A.N., Vinogradova I.D., Ugriumova G.A., Solovyev A.I., Esmagambetov I.B., Tukhvatulin A.I., Logunov D.Y., Naroditsky B.S., Shcheblyakov D.V. (2019). Camelid VHHs Fused to Human Fc Fragments Provide Long Term Protection Against Botulinum Neurotoxin A in Mice. Toxins.

[57] Altunbulakli C., Reiger M., Neumann A.U., Garzorz-Stark N., Fleming M., Huelpuesch C., Castro-Giner F., Eyerich K., Akdis C.A., Traidl-Hoffmann C. (2018). Relations between Epidermal Barrier Dysregulation and Staphylococcus Species–Dominated Microbiome Dysbiosis in Patients with Atopic Dermatitis. J. Allergy Clin. Immunol..

[58] Fyhrquist N., Muirhead G., Prast-Nielsen S., Jeanmougin M., Olah P., Skoog T., Jules-Clement G., Feld M., Barrientos-Somarribas M., Sinkko H. (2019). Microbe-Host Interplay in Atopic Dermatitis and Psoriasis. Nat. Commun..

[59] Chng K.R., Tay A.S.L., Li C., Ng A.H.Q., Wang J., Suri B.K., Matta S.A., McGovern N., Janela B., Wong X.F.C.C. (2016). Whole Metagenome Profiling Reveals Skin Microbiome-Dependent Susceptibility to Atopic Dermatitis Flare. Nat. Microbiol..

[60] Koh L.F., Ong R.Y., Common J.E. (2022). Skin Microbiome of Atopic Dermatitis. Allergol. Int..

[61] Rauer L., Reiger M., Bhattacharyya M., Brunner P.M., Krueger J.G., Guttman-Yassky E., Traidl-Hoffmann C., Neumann A.U. (2023). Skin Microbiome and Its Association with Host Cofactors in Determining Atopic Dermatitis Severity. J. Eur. Acad. Dermatol. Venereol..

[62] Simpson E.L., Villarreal M., Jepson B., Rafaels N., David G., Hanifin J., Taylor P., Boguniewicz M., Yoshida T., De Benedetto A. (2018). Patients with Atopic Dermatitis Colonized with *Staphylococcus aureus* Have a Distinct Phenotype and Endotype. J. Investig. Dermatol..

[63] Hülpüsch C., Tremmel K., Hammel G., Bhattacharyya M., de Tomassi A., Nussbaumer T., Neumann A.U., Reiger M., Traidl-Hoffmann C. (2020). Skin PH–Dependent *Staphylococcus aureus* Abundance as Predictor for Increasing Atopic Dermatitis Severity. Allergy.

[64] Tauber M., Balica S., Hsu C.-Y., Jean-Decoster C., Lauze C., Redoules D., Viodé C., Schmitt A.-M., Serre G., Simon M. (2016). *Staphylococcus aureus* Density on Lesional and Nonlesional Skin Is Strongly Associated with Disease Severity in Atopic Dermatitis. J. Allergy Clin. Immunol..

[65] Cho S.-H., Strickland I., Tomkinson A., Fehringer A.P., Gelfand E.W., Leung D.Y.M. (2001). Preferential Binding of *Staphylococcus aureus* to Skin Sites of Th2-Mediated Inflammation in a Murine Model. J. Investig. Dermatol..

[66] Miajlovic H., Fallon P.G., Irvine A.D., Foster T.J. (2010). Effect of Filaggrin Breakdown Products on Growth of and Protein Expression by *Staphylococcus aureus*. J. Allergy Clin. Immunol..

[67] Cabanillas B., Novak N. (2016). Atopic Dermatitis and Filaggrin. Curr. Opin. Immunol..

[68] Leung D.Y.M., Guttman-Yassky E. (2014). Deciphering the Complexities of Atopic Dermatitis: Shifting Paradigms in Treatment Approaches. J. Allergy Clin. Immunol..

[69] Salamzade R., Swaney M.H., Kalan L.R. (2023). Comparative Genomic and Metagenomic Investigations of the *Corynebacterium tuberculostearicum* Species Complex Reveals Potential Mechanisms Underlying Associations To Skin Health and Disease. Microbiol. Spectr..

[70] Edslev S.M., Olesen C.M., Nørreslet L.B., Ingham A.C., Iversen S., Lilje B., Clausen M.-L., Jensen J.S., Stegger M., Agner T. (2021). Staphylococcal Communities on Skin Are Associated with Atopic Dermatitis and Disease Severity. Microorganisms.

[71] Bjerre R.D., Holm J.B., Palleja A., Sølberg J., Skov L., Johansen J.D. (2021). Skin Dysbiosis in the Microbiome in Atopic Dermatitis Is Site-Specific and Involves Bacteria, Fungus and Virus. BMC Microbiol..

[72] Han S.H., Cheon H.I., Hur M.S., Kim M.J., Jung W.H., Lee Y.W., Choe Y.B., Ahn K.J. (2018). Analysis of the Skin Mycobiome in Adult Patients with Atopic Dermatitis. Exp. Dermatol..

[73] Zhang E., Tanaka T., Tajima M., Tsuboi R., Nishikawa A., Sugita T. (2011). Characterization of the Skin Fungal Microbiota in Patients with Atopic Dermatitis and in Healthy Subjects. Microbiol. Immunol..

[74] Selander C., Zargari A., Möllby R., Rasool O., Scheynius A. (2006). Higher PH Level, Corresponding to That on the Skin of Patients with Atopic Eczema, Stimulates the Release of *Malassezia sympodialis* Allergens. Allergy.

[75] Matricardi P.M., Kleine-Tebbe J., Hoffmann H.J., Valenta R., Hilger C., Hofmaier S., Aalberse R.C., Agache I., Asero R., Ballmer-Weber B. (2016). EAACI Molecular Allergology User’s Guide. Pediatr. Allergy Immunol..

[76] Glatz M., Buchner M., Bartenwerffer W., Schmid-Grendelmeier P., Worm M., Hedderich J., Fölster-Holst R. (2015). *Malassezia* spp.-Specific Immunoglobulin E Level Is a Marker for Severity of Atopic Dermatitis in Adults. Acta Derm. Venereol..

[77] Selander C., Engblom C., Nilsson G., Scheynius A., Andersson C.L. (2009). TLR2/MyD88-Dependent and -Independent Activation of Mast Cell IgE Responses by the Skin Commensal Yeast *Malassezia sympodialis*. J. Immunol..

[78] Hiragun T., Ishii K., Hiragun M., Suzuki H., Kan T., Mihara S., Yanase Y., Bartels J., Schröder J.-M., Hide M. (2013). Fungal Protein MGL_1304 in Sweat Is an Allergen for Atopic Dermatitis Patients. J. Allergy Clin. Immunol..

[79] Sparber F., De Gregorio C., Steckholzer S., Ferreira F.M., Dolowschiak T., Ruchti F., Kirchner F.R., Mertens S., Prinz I., Joller N. (2019). The Skin Commensal Yeast *Malassezia* Triggers a Type 17 Response That Coordinates Anti-Fungal Immunity and Exacerbates Skin Inflammation. Cell Host Microbe.

[80] Bitschar K., Sauer B., Focken J., Dehmer H., Moos S., Konnerth M., Schilling N.A., Grond S., Kalbacher H., Kurschus F.C. (2019). Lugdunin Amplifies Innate Immune Responses in the Skin in Synergy with Host- and Microbiota-Derived Factors. Nat. Commun..

[81] Suwarsa O., Hazari M.N., Dharmadji H.P., Dwiyana R.F., Effendi R.M.R.A., Hidayah R.M.N., Avriyanti E., Gunawan H., Sutedja E. (2021). A Pilot Study: Composition and Diversity of 16S RRNA Based Skin Bacterial Microbiome in Indonesian Atopic Dermatitis Population. Clin. Cosmet. Investig. Dermatol..

[82] Schmid B., Künstner A., Fähnrich A., Bersuch E., Schmid-Grendelmeier P., Busch H., Glatz M., Bosshard P.P. (2022). Dysbiosis of Skin Microbiota with Increased Fungal Diversity Is Associated with Severity of Disease in Atopic Dermatitis. J. Eur. Acad. Dermatol. Venereol..

[83] Kim S., Suh D.H., Lee S., Kim H.S., Cho S.H., Woo Y.R. (2024). Associations Between Skin Microbiome and Metabolome in the Pathogenesis of Atopic Dermatitis Patients With Scalp Involvement. Allergy Asthma Immunol. Res..

[84] Sugumaran D., Yong A.C.H., Stanslas J. (2024). Advances in Psoriasis Research: From Pathogenesis to Therapeutics. Life Sci..

[85] Vičić M., Kaštelan M., Brajac I., Sotošek V., Massari L.P. (2021). Current Concepts of Psoriasis Immunopathogenesis. Int. J. Mol. Sci..

[86] Assarsson M., Söderman J., Dienus O., Seifert O. (2020). Significant Differences in the Bacterial Microbiome of the Pharynx and Skin in Patients with Psoriasis Compared with Healthy Controls. Acta Derm. Venereol..

[87] Wei J., Zhu J., Xu H., Zhou D., Elder J.T., Tsoi L.C., Patrick M.T., Li Y. (2022). Alcohol Consumption and Smoking in Relation to Psoriasis: A Mendelian Randomization Study. Br. J. Dermatol..

[88] Zou X., Zou X., Gao L., Zhao H. (2024). Gut Microbiota and Psoriasis: Pathogenesis, Targeted Therapy, and Future Directions. Front. Cell Infect. Microbiol..

[89] Chen L., Li J., Zhu W., Kuang Y., Liu T., Zhang W., Chen X., Peng C. (2020). Skin and Gut Microbiome in Psoriasis: Gaining Insight Into the Pathophysiology of It and Finding Novel Therapeutic Strategies. Front. Microbiol..

[90] Hashiguchi Y., Yabe R., Chung S.-H., Murayama M.A., Yoshida K., Matsuo K., Kubo S., Saijo S., Nakamura Y., Matsue H. (2018). IL-36α from Skin-Resident Cells Plays an Important Role in the Pathogenesis of Imiquimod-Induced Psoriasiform Dermatitis by Forming a Local Autoamplification Loop. J. Immunol..

[91] Valdimarsson H., Baker B.S., Jónsdóttir I., Powles A., Fry L. (1995). Psoriasis: A T-Cell-Mediated Autoimmune Disease Induced by Streptococcal Superantigens?. Immunol. Today.

[92] Leung D.Y., Travers J.B., Giorno R., Norris D.A., Skinner R., Aelion J., Kazemi L.V., Kim M.H., Trumble A.E., Kotb M. (1995). Evidence for a Streptococcal Superantigen-Driven Process in Acute Guttate Psoriasis. J. Clin. Investig..

[93] Gao Z., Tseng C., Strober B.E., Pei Z., Blaser M.J. (2008). Substantial Alterations of the Cutaneous Bacterial Biota in Psoriatic Lesions. PLoS ONE.

[94] Fahlén A., Engstrand L., Baker B.S., Powles A., Fry L. (2012). Comparison of Bacterial Microbiota in Skin Biopsies from Normal and Psoriatic Skin. Arch. Dermatol. Res..

[95] Alekseyenko A.V., Perez-Perez G.I., De Souza A., Strober B., Gao Z., Bihan M., Li K., Methé B.A., Blaser M.J. (2013). Community Differentiation of the Cutaneous Microbiota in Psoriasis. Microbiome.

[96] Drago L., De Grandi R., Altomare G., Pigatto P., Rossi O., Toscano M. (2016). Skin Microbiota of First Cousins Affected by Psoriasis and Atopic Dermatitis. Clin. Mol. Allergy.

[97] Tett A., Pasolli E., Farina S., Truong D.T., Asnicar F., Zolfo M., Beghini F., Armanini F., Jousson O., De Sanctis V. (2017). Unexplored Diversity and Strain-Level Structure of the Skin Microbiome Associated with Psoriasis. NPJ Biofilms Microbiomes.

[98] Chang H.-W., Yan D., Singh R., Liu J., Lu X., Ucmak D., Lee K., Afifi L., Fadrosh D., Leech J. (2018). Alteration of the Cutaneous Microbiome in Psoriasis and Potential Role in Th17 Polarization. Microbiome.

[99] Assarsson M., Duvetorp A., Dienus O., Söderman J., Seifert O. (2018). Significant Changes in the Skin Microbiome in Patients with Chronic Plaque Psoriasis after Treatment with Narrowband Ultraviolet B. Acta Derm. Venereol..

[100] Stehlikova Z., Kostovcik M., Kostovcikova K., Kverka M., Juzlova K., Rob F., Hercogova J., Bohac P., Pinto Y., Uzan A. (2019). Dysbiosis of Skin Microbiota in Psoriatic Patients: Co-Occurrence of Fungal and Bacterial Communities. Front. Microbiol..

[101] Kayıran M.A., Sahin E., Koçoğlu E., Sezerman O.U., Gürel M.S., Karadağ A.S. (2022). Is Cutaneous Microbiota a Player in Disease Pathogenesis? Comparison of Cutaneous Microbiota in Psoriasis and Seborrheic Dermatitis with Scalp Involvement. Indian. J. Dermatol. Venereol. Leprol..

[102] Ruuskanen M.O., Vats D., Potbhare R., RaviKumar A., Munukka E., Ashma R., Lahti L. (2022). Towards Standardized and Reproducible Research in Skin Microbiomes. Environ. Microbiol..

[103] Perugini P., Grignani C., Condrò G., van der Hoeven H., Ratti A., Mondelli A., Colpani A., Bleve M. (2023). Skin Microbiota: Setting up a Protocol to Evaluate a Correlation between the Microbial Flora and Skin Parameters. Biomedicines.

[104] Quan C., Chen X.-Y., Li X., Xue F., Chen L.-H., Liu N., Wang B., Wang L.-Q., Wang X.-P., Yang H. (2020). Psoriatic Lesions Are Characterized by Higher Bacterial Load and Imbalance between *Cutibacterium* and *Corynebacterium*. J. Am. Acad. Dermatol..

[105] Hill C., Guarner F., Reid G., Gibson G.R., Merenstein D.J., Pot B., Morelli L., Canani R.B., Flint H.J., Salminen S. (2014). Expert Consensus Document: The International Scientific Association for Probiotics and Prebiotics Consensus Statement on the Scope and Appropriate Use of the Term Probiotic. Nat. Rev. Gastroenterol. Hepatol..

[106] Yu Y., Dunaway S., Champer J., Kim J., Alikhan A. (2020). Changing Our Microbiome: Probiotics in Dermatology. Br. J. Dermatol..

[107] Al-Ghazzewi F.H., Tester R.F. (2014). Impact of Prebiotics and Probiotics on Skin Health. Benef. Microbes.

[108] Ouwehand A.C., Båtsman A., Salminen S. (2003). Probiotics for the Skin: A New Area of Potential Application?. Lett. Appl. Microbiol..

[109] Lopes E.G., Moreira D.A., Gullón P., Gullón B., Cardelle-Cobas A., Tavaria F.K. (2017). Topical Application of Probiotics in Skin: Adhesion, Antimicrobial and Antibiofilm in Vitro Assays. J. Appl. Microbiol..

[110] Schön M.P. (2019). Adaptive and Innate Immunity in Psoriasis and Other Inflammatory Disorders. Front. Immunol..

[111] Guéniche A., Benyacoub J., Buetler T.M., Smola H., Blum S. (2006). Supplementation with Oral Probiotic Bacteria Maintains Cutaneous Immune Homeostasis after UV Exposure. Eur. J. Dermatol..

[112] Chae M., Kim B.J., Na J., Kim S.Y., Lee J.O., Kim Y.J., Lee E., Cho D., Roh J., Kim W. (2021). Antimicrobial Activity of *Lactiplantibacillus plantarum* APsulloc 331261 and APsulloc 331266 against Pathogenic Skin Microbiota. Front Biosci.

[113] Negi A., Kuo C.W., Hazam P.K., Yeh J.C., Lin W.C., Lou Y.C., Yu C.Y., Yu T.L., Lu T.M., Chen J.Y. (2025). Disruption of MRSA Biofilm and Virulence by Deep-Sea Probiotics: Impacts on Energy Metabolism and Host Antimicrobial Peptides. Probiotics Antimicrob. Proteins.

[114] Myles I.A., Earland N.J., Anderson E.D., Moore I.N., Kieh M.D., Williams K.W., Saleem A., Fontecilla N.M., Welch P.A., Darnell D.A. (2018). First-in-Human Topical Microbiome Transplantation with Roseomonas Mucosa for Atopic Dermatitis. JCI Insight.

[115] Nakatsuji T., Gallo R.L., Shafiq F., Tong Y., Chun K., Butcher A.M., Cheng J.Y., Hata T.R. (2021). Use of Autologous Bacteriotherapy to Treat *Staphylococcus aureus* in Patients with Atopic Dermatitis: A Randomized Double-Blind Clinical Trial. JAMA Dermatol..

[116] Cui H., Feng C., Zhang T., Martínez-Ríos V., Martorell P., Tortajada M., Cheng S., Cheng S., Duan Z. (2023). Effects of a Lotion Containing Probiotic Ferment Lysate as the Main Functional Ingredient on Enhancing Skin Barrier: A Randomized, Self-Control Study. Sci. Rep..

[117] Prince T., McBain A.J., O’Neill C.A. (2012). Lactobacillus Reuteri Protects Epidermal Keratinocytes from *Staphylococcus aureus*-Induced Cell Death by Competitive Exclusion. Appl. Environ. Microbiol..

[118] Park J.-Y., Lee J.Y., Hong S., Heo H., Lee H., Kim Y.G., Kim B.-K., Choi S.-I., Lee J. (2024). *Limosilactobacillus fermentum* MG5368 and *Lactiplantibacillus plantarum* MG989 Regulates Skin Health in UVB-Induced HaCaT Cells and Hairless Mice Model. Nutrients.

[119] Souak D., Barreau M., Courtois A., André V., Duclairoir Poc C., Feuilloley M.G.J., Gault M. (2021). Challenging Cosmetic Innovation: The Skin Microbiota and Probiotics Protect the Skin from UV-Induced Damage. Microorganisms.

[120] Jeong J.H., Lee C.Y., Chung D.K. (2016). Probiotic Lactic Acid Bacteria and Skin Health. Crit. Rev. Food Sci. Nutr..

[121] Callewaert C., Ravard Helffer K., Lebaron P. (2020). Skin Microbiome and Its Interplay with the Environment. Am. J. Clin. Dermatol..

[122] Rosignoli C., Thibaut de Ménonville S., Orfila D., Béal M., Bertino B., Aubert J., Mercenier A., Piwnica D. (2018). A Topical Treatment Containing Heat-Treated Lactobacillus Johnsonii NCC 533 Reduces *Staphylococcus aureus* Adhesion and Induces Antimicrobial Peptide Expression in an in Vitro Reconstructed Human Epidermis Model. Exp. Dermatol..

[123] Wang Y., Moon A., Huang J., Sun Y., Qiu H.J. (2022). Antiviral Effects and Underlying Mechanisms of Probiotics as Promising Antivirals. Front. Cell Infect. Microbiol..

[124] Zouboulis C.C., Fischer T.C., Wohlrab J., Barnard J., Alió A.B. (2009). Study of the Efficacy, Tolerability, and Safety of 2 Fixed-Dose Combination Gels in the Management of Acne Vulgaris. Cutis.

[125] Lebeer S., Oerlemans E.F.M., Claes I., Henkens T., Delanghe L., Wuyts S., Spacova I., van den Broek M.F.L., Tuyaerts I., Wittouck S. (2022). Selective Targeting of Skin Pathobionts and Inflammation with Topically Applied Lactobacilli. Cell Rep. Med..

[126] Gueniche A., Knaudt B., Schuck E., Volz T., Bastien P., Martin R., Röcken M., Breton L., Biedermann T. (2008). Effects of Nonpathogenic Gram-Negative Bacterium Vitreoscilla Filiformis Lysate on Atopic Dermatitis: A Prospective, Randomized, Double-Blind, Placebo-Controlled Clinical Study. Br. J. Dermatol..

[127] Guéniche A., Hennino A., Goujon C., Dahel K., Bastien P., Martin R., Jourdain R., Breton L. (2006). Improvement of Atopic Dermatitis Skin Symptoms by Vitreoscilla Filiformis Bacterial Extract. Eur. J. Dermatol..

[128] Nguyen A.T., Kim M., Kim Y.E., Kim H., Lee S., Lee Y., Kim K.Y. (2023). MSF Enhances Human Antimicrobial Peptide β-Defensin (HBD2 and HBD3) Expression and Attenuates Inflammation via the NF- κ B and P38 Signaling Pathways. Molecules.

[129] Chen Y.H., Wu C.S., Chao Y.H., Lin C.C., Tsai H.Y., Li Y.R., Chen Y.Z., Tsai W.H., Chen Y.K. (2017). *Lactobacillus pentosus* GMNL-77 Inhibits Skin Lesions in Imiquimod-Induced Psoriasis-like Mice. J. Food Drug Anal..

[130] Asadullah K., Sterry W., Stephanek K., Jasulaitis D., Leupold M., Audring H., Volk H.D., Döcke W.D. (1998). IL-10 Is a Key Cytokine in Psoriasis: Proof of Principle by IL-10 Therapy: A New Therapeutic Approach. J. Clin. Investig..

[131] Tsai W.H., Chou C.H., Chiang Y.J., Lin C.G., Lee C.H. (2021). Regulatory Effects of *Lactobacillus plantarum*-GMNL6 on Human Skin Health by Improving Skin Microbiome. Int. J. Med. Sci..

[132] Gueniche A., Philippe D., Bastien P., Reuteler G., Blum S., Castiel-Higounenc I., Breton L., Benyacoub J. (2014). Randomised Double-Blind Placebo-Controlled Study of the Effect of *Lactobacillus paracasei* NCC 2461 on Skin Reactivity. Benef. Microbes.

[133] Gudadappanavar A., Hombal P., Timashetti S., Javali S. (2017). Influence of *Lactobacillus acidophilus* and *Lactobacillus plantarum* on Wound Healing in Male Wistar Rats—An Experimental Study. Int. J. Appl. Basic. Med. Res..

[134] Saha U.B., Saroj S.D. (2022). Lactic Acid Bacteria: Prominent Player in the Fight against Human Pathogens. Expert Rev. Anti -Infect. Ther..

[135] Öhnstedt E., Tomenius H.L., Frank P., Roos S., Vågesjö E., Phillipson M. (2022). Accelerated Wound Healing in Minipigs by On-Site Production and Delivery of CXCL12 by Transformed Lactic Acid Bacteria. Pharmaceutics.

[136] Li Y., Tang X., Liu Y., Chen X., Wang L., Su Y., He W., Li J., Huang Q., Wu A. (2025). Advanced Wound Healing with Chitosan Hydrogels Incorporating Metabolites from Whale-Derived *Lactiplantibacillus plantarum* HJ-S2. Front. Mater..

[137] Peral M.C., Rachid M.M., Gobbato N.M., Huaman Martinez M.A., Valdez J.C. (2010). Interleukin-8 Production by Polymorphonuclear Leukocytes from Patients with Chronic Infected Leg Ulcers Treated with *Lactobacillus plantarum*. Clin. Microbiol. Infect..

[138] Peral M.C., Huaman Martinez M.A., Valdez J.C. (2009). Bacteriotherapy with *Lactobacillus plantarum* in Burns. Int. Wound J..

[139] Valdéz J.C., Peral M.C., Rachid M., Santana M., Perdigón G. (2005). Interference of *Lactobacillus plantarum* with *Pseudomonas aeruginosa* in Vitro and in Infected Burns: The Potential Use of Probiotics in Wound Treatment. Clin. Microbiol. Infect..

[140] Hong Y.F., Lee H.Y., Jung B.J., Jang S., Chung D.K., Kim H. (2015). Lipoteichoic Acid Isolated from *Lactobacillus plantarum* Down-Regulates UV-Induced MMP-1 Expression and up-Regulates Type I Procollagen through the Inhibition of Reactive Oxygen Species Generation. Mol. Immunol..

[141] Lee K., Kim H.J., Kim S.A., Park S.D., Shim J.J., Lee J.L. (2021). Exopolysaccharide from *Lactobacillus plantarum* Hy7714 Protects against Skin Aging through Skin–Gut Axis Communication. Molecules.

[142] Kim M.S., Lee M., Oh H., Seo W., Kim G.S., Ban O.H., Shin M., Jung Y.H., Yang J. (2021). Enhanced Ceramides Production by *Lactobacillus rhamnosus* IDCC 3201 and Its Proposed Mechanism. Appl. Biol. Chem..

[143] Sambanthamoorthy K., Feng X., Patel R., Patel S., Paranavitana C. (2014). Antimicrobial and Antibiofilm Potential of Biosurfactants Isolated from Lactobacilli against Multi-Drug-Resistant Pathogens. BMC Microbiol..

[144] Qi X., Xiao Y., Zhang X., Zhu Z., Zhang H., Wei J., Zhao Z., Li J., Chen T. (2024). Probiotics Suppress LL37 Generated Rosacea-like Skin Inflammation by Modulating the TLR2/MyD88/NF-ΚB Signaling Pathway. Food Funct..

[145] Ahn S.H., Yoon W., Lee S.Y., Shin H.S., Lim M.Y., Nam Y.D., Yoo Y. (2020). Effects of *Lactobacillus pentosus* in Children with Allergen-Sensitized Atopic Dermatitis. J. Korean Med. Sci..

[146] Groeger D., O’Mahony L., Murphy E.F., Bourke J.F., Dinan T.G., Kiely B., Shanahan F., Quigley E.M.M. (2013). Bifidobacterium Infantis 35624 Modulates Host Inflammatory Processes beyond the Gut. Gut Microbes.

[147] Jiraskova Zakostelska Z., Reiss Z., Tlaskalova-Hogenova H., Rob F. (2023). Paradoxical Reactions to Anti-TNFα and Anti-IL-17 Treatment in Psoriasis Patients: Are Skin and/or Gut Microbiota Involved?. Dermatol. Ther..

[148] Buhaș M.C., Candrea R., Gavrilaș L.I., Miere D., Tătaru A., Boca A., Cătinean A. (2023). Transforming Psoriasis Care: Probiotics and Prebiotics as Novel Therapeutic Approaches. Int. J. Mol. Sci..

[149] Habeebuddin M., Karnati R.K., Shiroorkar P.N., Nagaraja S., Asdaq S.M.B., Anwer M.K., Fattepur S. (2022). Topical Probiotics: More Than a Skin Deep. Pharmaceutics.

[150] Ray Mohapatra A., Harikrishnan A., Lakshmanan D., Jeevaratnam K. (2022). Targeting *Staphylococcus aureus* and Its Biofilms with Novel Antibacterial Compounds Produced by *Lactiplantibacillus plantarum* SJ33. Arch. Microbiol..

[151] Wang Y., Kuo S., Shu M., Yu J., Huang S., Dai A., Two A., Gallo R.L., Huang C.M. (2014). Staphylococcus Epidermidis in the Human Skin Microbiome Mediates Fermentation to Inhibit the Growth of Propionibacterium Acnes: Implications of Probiotics in Acne Vulgaris. Appl. Microbiol. Biotechnol..

[152] Kober M.M., Bowe W.P. (2015). The Effect of Probiotics on Immune Regulation, Acne, and Photoaging. Int. J. Womens. Dermatol..

[153] Mintoff D., Borg I., Pace N.P. (2021). The Clinical Relevance of the Microbiome in Hidradenitis Suppurativa: A Systematic Review. Vaccines.

[154] Frew J.W., Hawkes J.E., Krueger J.G. (2019). Topical, Systemic and Biologic Therapies in Hidradenitis Suppurativa: Pathogenic Insights by Examining Therapeutic Mechanisms. Ther. Adv. Chronic. Dis..

[155] Dessinioti C., Katsambas A. (2024). The Microbiome and Acne: Perspectives for Treatment. Dermatol. Ther..

[156] Sánchez-Pellicer P., Eguren-Michelena C., García-Gavín J., Llamas-Velasco M., Navarro-Moratalla L., Núñez-Delegido E., Agüera-Santos J., Navarro-López V. (2024). Rosacea, Microbiome and Probiotics: The Gut-Skin Axis. Front. Microbiol..

[157] Daou H., Paradiso M., Hennessy K., Seminario-Vidal L. (2021). Rosacea and the Microbiome: A Systematic Review. Dermatol. Ther..

[158] Liegenfeld S.C., Stenzel S., Rembe J.-D., Dittmer M., Ramos P., Stuermer E.K. (2025). Pathogenic and Non-Pathogenic Microbes in the Wound Microbiome—How to Flip the Switch. Microbiol. Res..

[159] Burns E.M., Ahmed H., Isedeh P.N., Kohli I., Van Der Pol W., Shaheen A., Muzaffar A.F., Al-Sadek C., Foy T.M., Abdelgawwad M.S. (2019). Ultraviolet Radiation, Both UVA and UVB, Influences the Composition of the Skin Microbiome. Exp. Dermatol..

[160] Calvo M.J., Navarro C., Durán P., Galan-Freyle N.J., Parra Hernández L.A., Pacheco-Londoño L.C., Castelanich D., Bermúdez V., Chacin M. (2024). Antioxidants in Photoaging: From Molecular Insights to Clinical Applications. Int. J. Mol. Sci..

[161] Świerczewska Z., Lewandowski M., Surowiecka A., Barańska-Rybak W. (2022). Microbiome in Hidradenitis Suppurativa—What We Know and Where We Are Heading. Int. J. Mol. Sci..

